# Accumulation of Succinyl Coenzyme A Perturbs the Methicillin-Resistant *Staphylococcus aureus* (MRSA) Succinylome and Is Associated with Increased Susceptibility to Beta-Lactam Antibiotics

**DOI:** 10.1128/mBio.00530-21

**Published:** 2021-06-29

**Authors:** Christopher Campbell, Claire Fingleton, Merve S. Zeden, Emilio Bueno, Laura A. Gallagher, Dhananjay Shinde, Jongsam Ahn, Heather M. Olson, Thomas L. Fillmore, Joshua N. Adkins, Fareha Razvi, Kenneth W. Bayles, Paul D. Fey, Vinai C. Thomas, Felipe Cava, Geremy C. Clair, James P. O’Gara

**Affiliations:** a Microbiology, School of Natural Sciences, National University of Ireland, Galway, Ireland; b MIMS-Molecular Infection Medicine Sweden, Molecular Biology Department, Umeå University, Umeå, Sweden; c Department of Pathology and Microbiology, University of Nebraska Medical Center, Omaha, Nebraska, USA; d Biological Sciences Division, Pacific Northwest National Laboratory, Richland, Washington, USA; New York University School of Medicine

**Keywords:** MRSA, TCA cycle, antibiotic resistance, beta-lactams, succinyl-CoA, succinylome

## Abstract

Penicillin binding protein 2a (PBP2a)-dependent resistance to β-lactam antibiotics in methicillin-resistant Staphylococcus aureus (MRSA) is regulated by the activity of the tricarboxylic acid (TCA) cycle via a poorly understood mechanism. We report that mutations in *sucC* and *sucD*, but not other TCA cycle enzymes, negatively impact β-lactam resistance without changing PBP2a expression. Increased intracellular levels of succinyl coenzyme A (succinyl-CoA) in the *sucC* mutant significantly perturbed lysine succinylation in the MRSA proteome. Suppressor mutations in *sucA* or *sucB*, responsible for succinyl-CoA biosynthesis, reversed *sucC* mutant phenotypes. The major autolysin (Atl) was the most succinylated protein in the proteome, and increased Atl succinylation in the *sucC* mutant was associated with loss of autolytic activity. Although PBP2a and PBP2 were also among the most succinylated proteins in the MRSA proteome, peptidoglycan architecture and cross-linking were unchanged in the *sucC* mutant. These data reveal that perturbation of the MRSA succinylome impacts two interconnected cell wall phenotypes, leading to repression of autolytic activity and increased susceptibility to β-lactam antibiotics.

## INTRODUCTION

Staphylococcus aureus can establish infections in humans in a wide range of metabolic niches due to several signal transduction pathways, as well as virulence genes encoded in its genome. Significant advances have been made in the study of bacterial virulence factors and their functions in human disease. However, we have only just begun to understand the metabolic pathways required for bacterial proliferation in the host and their contribution to antibiotic resistance.

The *blaZ*-encoded β-lactamase hydrolyses the β-lactam ring in penicillin, conferring penicillin resistance in S. aureus (1). Methicillin resistance is mediated by alternative penicillin-binding protein 2a (PBP2a), encoded by *mecA* (2) located on a mobile genetic element, the staphylococcal chromosome cassette (SCC*mec*) (3, 4). PBP2a has low affinity for β-lactam antibiotics and thus is able to cross-link peptidoglycan (PG) strands even in the presence of β-lactam antibiotics and in this manner confers resistance (2). Methicillin-resistant S. aureus (MRSA) strains are resistant to methicillin, as well as to all the other β-lactam antibiotics (1, 2), consequently making infections with MRSA difficult to treat.

β-Lactam resistance in S. aureus is typically expressed heterogeneously within a given population (5). The majority of cells within a heterogeneous population exhibit susceptible or borderline susceptible resistance to β-lactams. A subpopulation of approximately 0.1% can survive antibiotic treatment and, upon reexposure to the antibiotic, a homogeneously resistant population emerges (5). The mechanisms underpinning this switch from heterogeneous resistance (HeR) to homogenous resistance (HoR) are associated with accessory mutations outside *mecA* (6). High-level β-lactam resistance is accompanied by significant energy demands that impose a fitness cost on the cell (7, 8).

The activity of the tricarboxylic acid (TCA) cycle is an important source for the generation of NADH, and therefore membrane potential, during aerobic respiration. In addition, the TCA cycle generates metabolic intermediates that are then used in various other pathways in the cell. The genes encoding enzymes for the TCA cycle are repressed when preferred nutrients, such as glucose, remain available in the surrounding medium (9). Once post-exponential growth is reached and glucose is depleted from the medium, TCA cycle gene expression is derepressed in S. aureus (10–12). The activity of the TCA cycle has previously been linked to β-lactam resistance in S. epidermidis, where a dysfunctional TCA cycle is common among clinical isolates and is associated with alterations in the cell envelope and increased tolerance to β-lactams (13). In S. aureus, TCA cycle activity regulates ATP levels, which controls tolerance to several antibiotics, including β-lactams (12). Increased TCA cycle activity to fuel cell wall biosynthesis has also been shown to accompany mutations that enable the transition from the HeR to HoR phenotypes (14, 15). Furthermore, disruption of the TCA cycle via mutations in *acn* and *citZ* has been reported to block the production of HoR mutants (14, 15).

Post-translational modification (PTM) of proteins is one of the most effective mechanisms in diversifying protein function and regulation (16). PTMs can change the charge and structure of a protein, thus affecting activity, as well as the ability to interact with other proteins/binding partners (17, 18). Lysine is a basic residue that is critical for protein structure and function (19). The side chain of lysine in particular can be modified by a variety of PTMs, including phosphorylation (20), succinylation (21–23), ubiquitination (24), methylation (25), acetylation (16, 26), and lipoylation (27). Relatively little is known about PTMs in S. aureus metabolism and antibiotic resistance, and advances in our understanding of these systems will generate new insights into fundamental cellular processes and virulence mechanisms and potentially identify new therapeutic targets.

In the present study, we report that mutations in the succinyl coenzyme A (succinyl-CoA) synthetase genes, *sucC* and *sucD*, lead to increased susceptibility to β-lactam antibiotics in MRSA strain JE2. The impact of these mutations on growth was measured and compared to other TCA cycle mutants. The relative intracellular concentrations of metabolites from the pyruvate node of glycolysis and the TCA cycle were measured, and PG architecture and autolytic activity compared in the *sucC* mutant and wild-type JE2. We describe the first profile of the lysine succinylome in MRSA and the impact of the *sucC* mutation on the global succinylome. Our data reveal that increased accumulation of succinyl-CoA from the TCA cycle increases susceptibility to β-lactam antibiotics and reduces autolytic activity via perturbation of global lysine succinylation in MRSA.

## RESULTS

### TCA cycle genes *sucC* and *sucD* control resistance to β-lactam antibiotics in MRSA.

**FIG 1:**
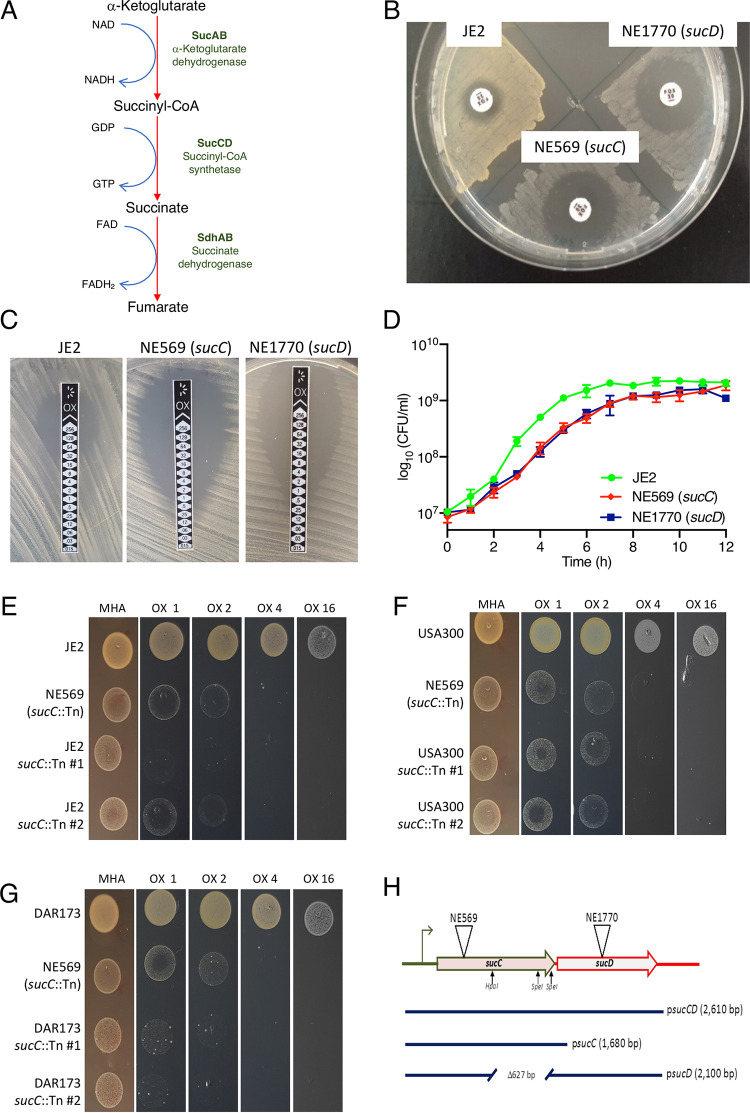
Mutation of *sucC* or *sucD* increases β-lactam susceptibility in MRSA and impairs growth in MHB. (A) *sucCD*-encoded succinyl-CoA synthetase catalyzes the conversion of succinyl-CoA to succinate in the TCA cycle. *sucAB*-encoded α-ketoglutarate dehydrogenase catalyzes conversion of a-ketoglutarate to succinyl-CoA, and *sdhAB*-encoded succinate dehydrogenase converts succinate to fumarate. (B) Measurement of JE2, NE569, and NE1770 cefoxitin susceptibility by disk diffusion assay. (C) M.I.C.Evaluator measurement of oxacillin MICs for JE2, NE569, and NE1770. (D) Growth of JE2, NE569 (*sucC*), and NE1770 (*sucD*) in MHB (no antibiotic supplementation) at 37°C. CFU were enumerated at 1-h intervals for 12 h. The data are the average of three independent experiments, and error bars represent standard deviations. (E) JE2, NE569 (*sucC*::Tn), and JE2 *sucC*::Tn transductants 1 and 2 spot-inoculated onto MHA and MHA oxacillin (OX) at 1, 2, 4, and 16 µg/ml. (F) DAR173, NE569, and DAR173 *sucC*::Tn transductants 1 and 2 spot inoculated onto MHA and MHA OX at 1, 2, 4, and 16 µg/ml. (G) USA300, NE569, and USA300 *sucC*::Tn transductants 1 and 2 spot inoculated onto MHA and MHA OX at 1, 2, 4, and 16 µg/ml. These assays were repeated three times, and a representative image is shown. (H) Chromosomal organization of the *sucCD* locus, including the locations of transposon insertions in NE569 (*sucC*) and NE1770 (*sucD*). The parts of the *sucCD* operon carried on p*sucCD*, *psucC*, and ps*ucD* used in complementation experiments are indicated.

A screen of the Nebraska Transposon Mutant Library (NTML) (28) revealed that the TCA cycle mutants, NE569 (*sucC*::Tn) and NE1770 (*sucD*::Tn) were more susceptible to cefoxitin (Fig. 1A and B) and oxacillin (Fig. 1C; Table 1) compared to wild-type JE2. Other mutations in TCA cycle genes did not affect susceptibility to oxacillin (Table 1), specifically implicating *sucCD*-encoded succinyl-CoA synthetase in β-lactam resistance. The oxacillin MICs of the *sucC* and *sucD* mutants in Mueller-Hinton agar (MHA) were 2 to 4 µg/ml, compared to 32 to 64 µg/ml for JE2, as measured using M.I.C.Evaluator (Fig. 1C) and agar dilution assays (Table 1). Both mutants also exhibited reduced growth in Mueller-Hinton broth (MHB) in the absence of antibiotic (Fig. 1D). Phage 80α-mediated backcross of the *sucC*::Tn allele into JE2, USA300 FPR3757 (4), and clinical MRSA strain DAR173 (29, 30) was accompanied by increased susceptibility to oxacillin (Fig. 1E to G; Table 1). In addition, both NE569 and NE1770 were successfully complemented by the *sucCD* genes carried on plasmid pLI50 (Table 1). The *sucC* and *sucD* genes are separated by only 21 bp, suggesting that they are organized in an operon (Fig. 1H) and that the transposon insertion in *sucC* is likely to have a polar effect on *sucD* expression. Consistent with this, NE569 was complemented by p*sucCD* but not by plasmids carrying *sucC* (p*sucC*) or *sucD* (p*sucD*) alone (Table 1 and Fig. 1H). NE1770 was complemented by *psucCD* and partially complemented by p*sucD*, but not by p*sucC* (Table 1 and Fig. 1H).

**TABLE 1:** Oxacillin MICs of strains described in this study

Strain and relevant details	Oxacillin MIC (µg/ml)^*a*^
Wild type and *sucC* and *sucD* mutants	
JE2	32–64
NE569 (*sucC*::Tn)	2–4
NE1770 (*sucD*::Tn)	4
	
Complemented *sucC* and *sucD* mutants	
NE569 p*sucC*	0.5
NE569 p*sucD*	1
NE569 p*sucCD*	32
NE1770 p*sucC*	0.5
NE1770 p*sucD*	16
NE1770 p*sucCD*	32
	
Transduction of *sucC*::Tn into JE2, DAR173 and USA300	
JE2 *sucC*::Tn transductant 1	2–4
JE2 *sucC*::Tn transductant 2	2–4
DAR173	64
DAR173 *sucC*::Tn transductant 1	2–4
DAR173 *sucC*::Tn transductant 2	2–4
USA300	32–64
USA300 *sucC*::Tn transductant 1	4
USA300 *sucC*::Tn transductant 2	4
	
	
Other TCA cycle mutants	
NE547 (*sucA*)	32
NE1391 (*sucB*)	32
NE626 (*sdhA*)	32
NE808 (*sdhB*)	32
NE594 (*gltA*)	32
NE861 (*acn*)	32
NE491 (*icd*)	32
NE427 (*fumC*)	64
	
*sucC*, *sucA*, and *sdhA* mutants and *sucC* suppressor mutants	
*sucC*::Tn-Kan^r^	4
*sucC* *sucA* double mutant	64
*sucC* *sdhA* double mutant	8
*sucC* suppressor 1	64
*sucC* suppressor 2	64
*sucC* suppressor 3	64
*sucC* HoR and *sucC* *relA* double mutants	
*sucC* HoR1	>256
*sucC* HoR2	>256
NE1714 (*relA*)	>256
*sucC* *relA* double mutant	>256

a Measured by agar dilution assays.

### HoR mutants of NE569 (*sucC*) have mutations in *relA* and *relQ*.

Expression of the HoR phenotype is dependent on activation of the (p)ppGpp-mediated stringent response (31–34). SucCD, succinyl-CoA synthetase, produces GTP, which is a substrate for (p)ppGpp synthesis by the RelA/SpoT homolog (RSH), RelP, and RelQ, raising the possibility that the NE569 (*sucC*) or NE1770 (*sucD*) mutations may negatively affect (p)ppGpp production. To investigate the possible relationship between the impact of *sucCD* mutations and the stringent response, the ability of JE2, NE569, and NE1770 to produce HoR mutants on brain heart infusion (BHI) agar supplemented with oxacillin 100 µg/ml was compared. Two stable NE569 HoR mutants, designated *sucC* HoR1 and *sucC* HoR2 with oxacillin MICs >256 µg/ml (see Fig. S1A in the supplemental material), were genome sequenced. *sucC* HoR1 had a single nucleotide deletion, frameshift mutation in *relA* (Table 2; see also Fig. S1B), while *sucC* HoR2 had a nonsynonymous point mutation, resulting in an A_178_V substitution in RelQ (Table 2; see also Fig. S1C). The N-terminus of RelA contains (p)ppGpp hydrolase and synthase domains, while mutations in the C-terminal domain deregulate synthase activity (35–37). The NTML *relA* mutant, NE1714, contains a transposon insertion in the C-terminal domain and is associated with increased β-lactam resistance, presumably due to increased (p)ppGpp synthase activity (Table 1). To investigate the impact of the *relA*::Tn mutation on β-lactam susceptibility in NE569, a *relA sucC* double mutant was constructed. First, the erythromycin resistance (Erm^r^) marker in NE569 was swapped for a kanamycin resistance (Kan^r^) marker, as described in Materials and Methods, to generate *sucC*::Tn-Kan^r^. The *sucC*::Kan^r^ allele was then transduced into NE1714 using phage 80α. Similar to the *sucC* HoR mutants, the oxacillin MIC of the *relA sucC* double mutant was also >256 μg/ml (Table 1). RelQ contains a (p)ppGpp synthase domain only and has previously been implicated in adaptation to vancomycin- and ampicillin-induced cell wall stress (38). A *relQ* mutant is not available in the NTML, indicating that this gene may be essential and that the RelQ A_178_V substitution in *sucC* HoR2 may enhance ppGpp synthase activity to promote a HoR phenotype. Given that previous studies have demonstrated the role of ppGpp synthase mutations in the HoR phenotype (5, 38, 39), these data suggest that increased β-lactam susceptibility in the *sucCD* mutants is not related to impaired GTP/ppGpp production.

**TABLE 2:** Genomic changes in NE569 (*sucC*::Tn), *sucC* HoR1, *sucC* HoR2, and *sucC* suppressors 1, 2, and 3

Strain	Reference position^*a*^	Type^*b*^	Reference^*c*^	Allele^*d*^	Frequency (%)^*e*^	Avg quality^*f*^	Annotation(s)	aa change^*g*^
NE569 (*sucC*::Tn)	1247099–1247100	INS	TA	T-*Tn*-A	100	NA	*Bursa aurealis* transposon	NA
*sucC* HoR1	1741861	DEL	A		97.2	37.2	SAUSA300_RS08665, *relA*	Tyr_418_STOP
*sucC* HoR2	994827	SNV	C	T	98.4	35.5	SAUSA300_RS04880, *relQ*	Ala_178_Val
*sucC* suppressor 1	1247099–1247100	INS	TA	T-*Tn*-A	100	NA	*Bursa aurealis* transposon	NA
	528497	SNV	C	A	100	36.8	SAUSA300_RS02515, *tatD* family deoxyribonuclease	Cys_242_STOP
	1439926	SNV	G	T	100	37.9	SAUSA300_RS07105, *sucA*	Ser_9_STOP
*sucC* suppressor 2	1247099–1247100	INS	TA	T-*Tn*-A	100	NA	*Bursa aurealis* transposon	NA
	1319786	SNV	A	T	100	36.9	*hflX*	Ile_53_Phe
	1436058	SNV	A	G	100	33.1	SAUSA300_RS07100, *sucB*	Ile_361_Thr
*sucC* suppressor 3	1247099–1247100	INS	TA	T-*Tn*-A	100	NA	*Bursa aurealis* transposon	NA
	1436186–1436231	DEL	T-A	Δ46bp^*h*^	100	NA	SAUSA300_RS07100, *sucB*	NA

a Reference position: the position in the USA300 FPR3757 genome sequence (NC_007793.1).

b Type of mutation: SNV, single nucleotide variant; INS, insertion; DEL, nucleotide deletion.

c Nucleotide base in the USA300 FPR3757 reference genome.

d Allele refers to the nucleotide base at the same position in the sequenced strain.

e That is, the percent frequency at which SNV/INS was found in the sequenced strain.

f Average quality score. Higher scores, which take account of the average PHRED score and read coverage, indicate a smaller probability of error. A quality score of 30 represents an error rate of 1 in 1,000, with a corresponding call accuracy of 99.9%. NA, not applicable.

g The amino acid (aa) change denotes the resulting amino acid change in the protein found in the sequenced strain compared to the WT reference strain. NA, not applicable.

h A 46-bp deletion in *sucB* (AAATTAGCAATTTCTGCTTCGATTTCTGCAAAATTCTTTTTATC).

10.1128/mBio.00530-21.1 FIG S1 NE569 (*sucC*) HoR mutants contain RSH (*relA*) and *relQ* mutations. Download FIG S1, PDF file, 0.2 MB. Copyright © 2021 Campbell et al. 2021 Campbell et al. https://creativecommons.org/licenses/by/4.0/ This content is distributed under the terms of the Creative Commons Attribution 4.0 International license.

### Mutations in *sucA* and *sucB* reverse NE569 (*sucC*) mutant phenotypes.

**FIG 2:**
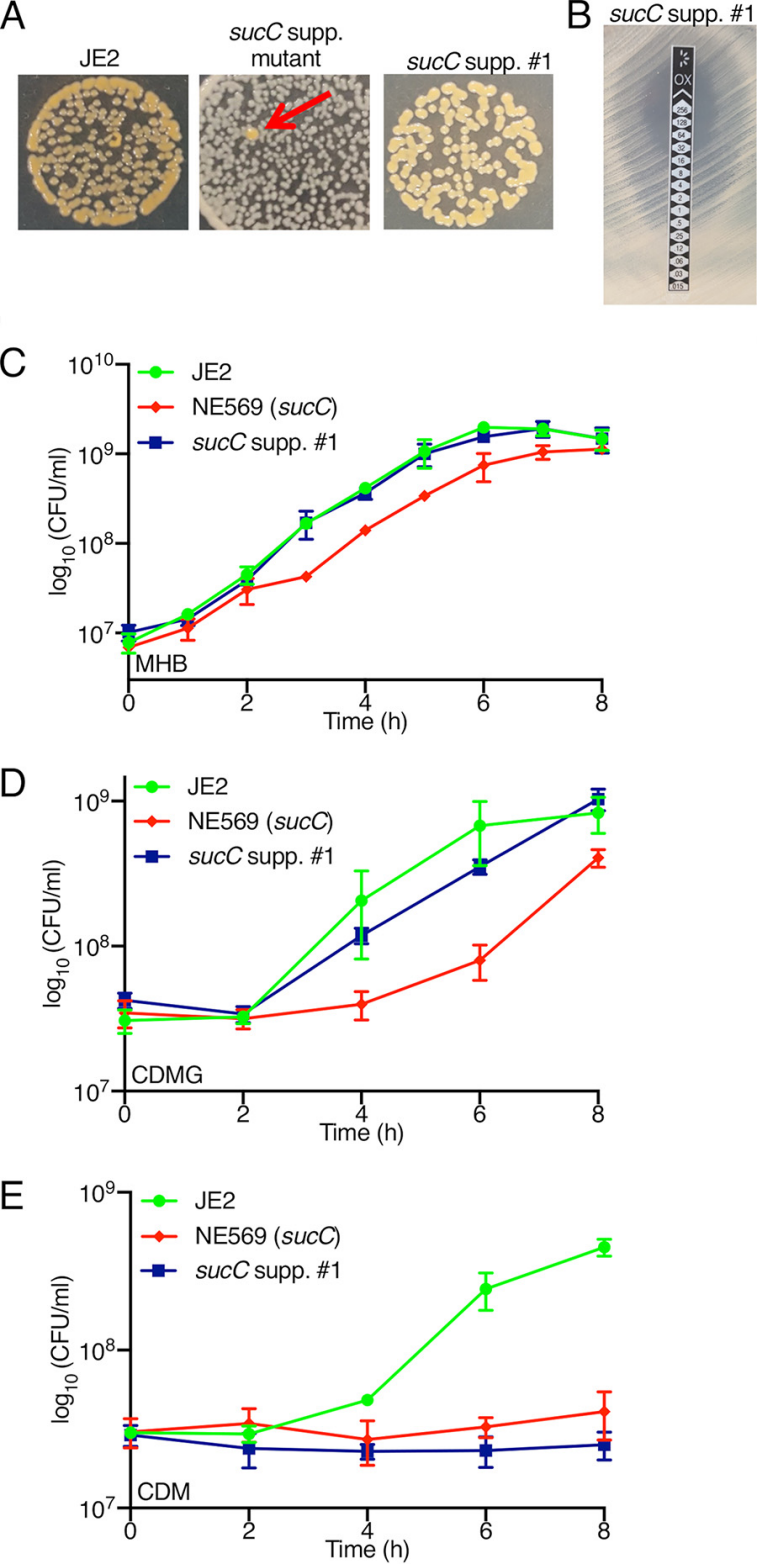
*sucC* suppressor mutation is accompanied by restoration of wild-type colony morphology, oxacillin resistance and growth in MHB and CDMG, but not CDM. (A) JE2, NE569 (*sucC*), and isolated *sucC* suppressor 1 grown on MHA for 48 h at 37°C. Red arrow indicates faster growing, more pigmented suppressor mutant of NE569. (B) M.I.C.Evaluator measurement of oxacillin MIC for *sucC* suppressor 1. Three independent measurements were performed and a representative image is shown. (C to E) Growth of JE2, *sucC* (NE569), and *sucC* suppressor 1 in MHB (C), CDMG (D), or CDM (E). Growth was measured by enumerating the number of CFU/ml at 2-h intervals in flask cultures. The data presented are the averages of at least three independent experiments, and error bars represent the standard deviations.

Faster-growing, more-pigmented suppressor mutants were readily observed among colonies of NE569 grown on MHA (Fig. 2A). Three of these suppressor mutants were isolated and comparison of their genomic DNA sequences to NE569 identified mutations in either *sucA* or *sucB* (Table 2). The *sucA* gene, which encodes 2-oxoglutarate dehydrogenase E1 subunit, is found in a two-gene operon with *sucB*, which encodes dihydrolipoyl succinyltransferase E2 subunit. Together, SucAB catalyze the synthesis of succinyl-CoA, the substrate for SucCD complex (Fig. 1A). A single nucleotide variant (SNV) leading to a Ser_9_STOP codon in *sucA* was present in *sucC* suppressor 1, *sucC* suppressor 2 had a SNV leading to a Ile_361_Thr substitution in SucB, and *sucC* suppressor 3 had a 46-bp deletion in *sucB*. The possible significance of additional SNVs in the *tatD* and *hflX* genes of *sucC* suppressors 1 and 2, respectively, is unknown. However, all three *sucC* suppressor mutants exhibited wild-type levels of oxacillin resistance (Table 1 and Fig. 2B), implicating the *sucA* and *sucB* mutations in this phenotype.

The *sucC* suppressor 1 mutant, which was chosen for more detailed analysis, also exhibited wild-type growth in MHB (Fig. 2C). Similarly, in chemically defined media supplemented with glucose (CDMG), the NE569 growth defect was reversed by the *sucA* suppressor mutation (Fig. 2D). However, both NE569 and *sucC* suppressor 1 were unable to grow in chemically defined media lacking glucose (CDM) (Fig. 2E), which is consistent with previous results showing growth in CDM is dependent on an intact TCA cycle (9).

**FIG 3:**
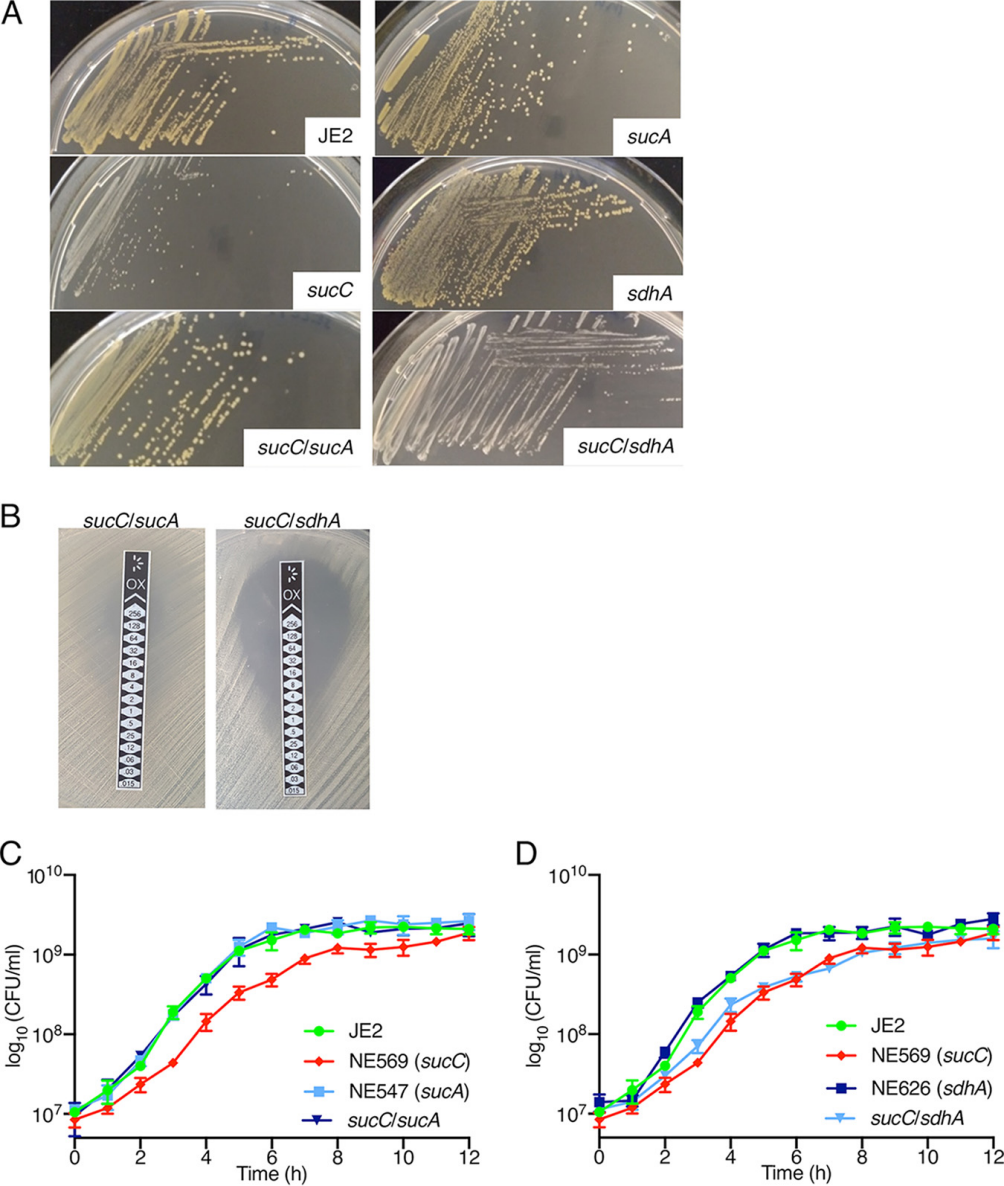
Mutation of *sucA*, but not *sdhA*, in the *sucC* background restores wild-type colony morphology, β-lactam resistance, and growth phenotypes. (A) Colony morphologies of JE2, NE569 (*sucC*), NE547 (*sucA*), NE626 (*sdhA*), *sucC* *sucA*, and *sucC* *sdhA* strains grown for 24 h on MHA. (B) M.I.C.Evaluator measurement of oxacillin MICs for *sucA sucC* and *sdhA sucC* strains. Three independent measurements were performed for each strain, and a representative image is shown. (C) Growth of JE2, NE569 (*sucC*), NE547 (*sucA*), and *sucA* *sucC* strains. (D) Growth of JE2, NE569, (*sucC*), NE626 (*sdhA*), and *sdhA* *sucC* strains. Growth experiments were performed in MHB at 37°C, and CFU/ml were enumerated at 1-h intervals for 12 h. All data presented are the average of three independent experiments, and error bars represent the standard deviations.

Extension of these experiments to NTML mutants revealed no change in oxacillin susceptibility in NE547 (*sucA*) and NE1391 (*sucB*) (Table 1). Using phage 80α to disrupt *sucA* in the *sucC* mutant restored wild-type colony morphology, oxacillin resistance, and growth (Fig. 3A to C). For control purposes, a *sucC sdhA* double mutant was also constructed. The succinate dehydrogenase complex SdhAB catalyzes the conversion of the SucCD product, succinate, to fumarate (Fig. 1A). Mutations in *sdhA* or *sdhB* did not impact susceptibility to oxacillin (Table 1) and the *sucC sdhA* double mutant exhibited the same colony morphology, oxacillin resistance, and growth characteristics as the *sucC* mutant (Fig. 3A, B, and D). Taken together, these data demonstrate that mutations in *sucA or sucB* overcome the β-lactam susceptibility and growth defects of the *sucC* mutant.

### Succinyl-CoA is significantly increased in the *sucC* mutant.

**FIG 4:**
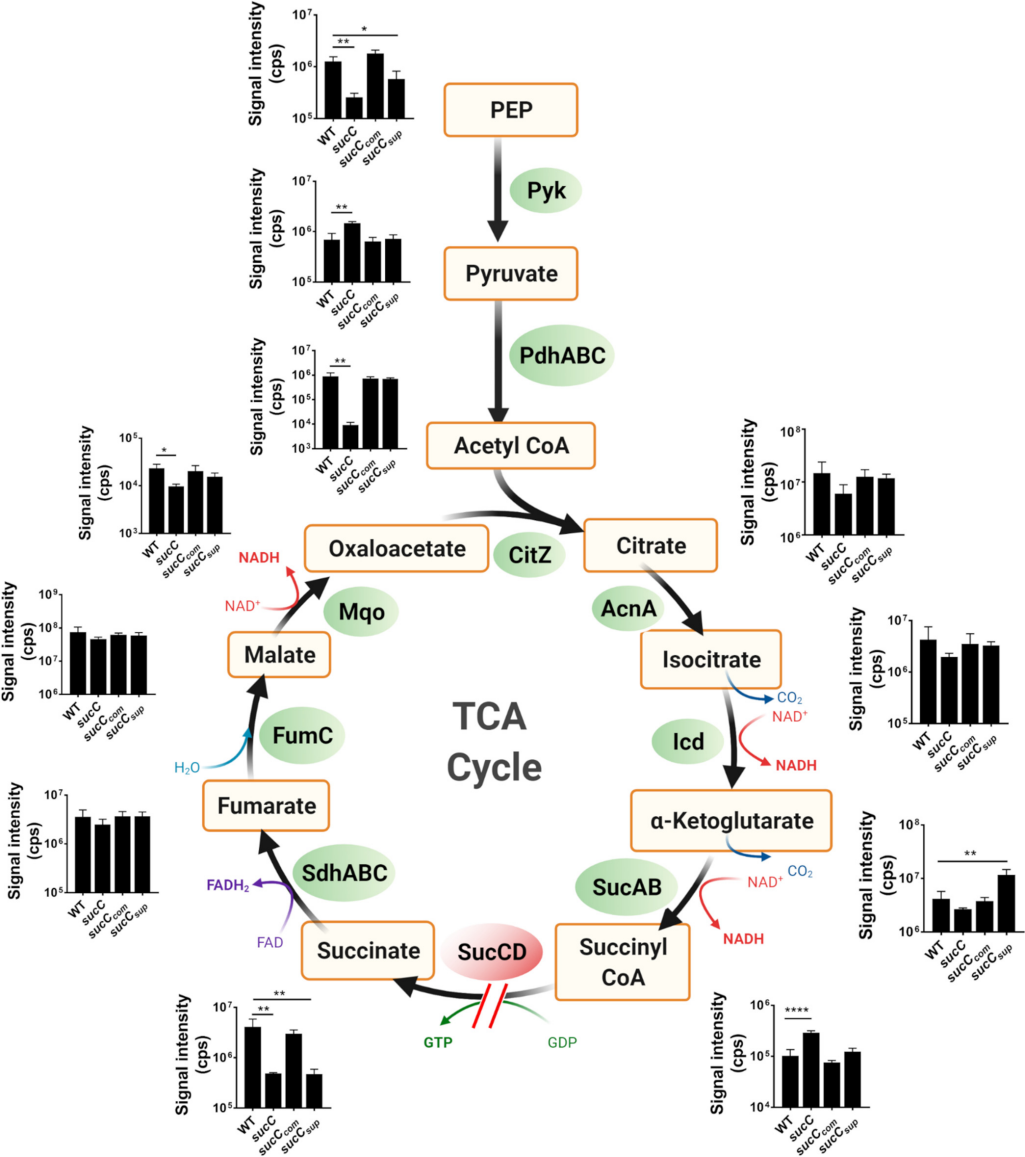
Mutation of *sucC* alters the central metabolism in S. aureus. JE2, NE569 (*sucC*), *sucC*_comp_ (NE569 p*sucCD*), and *sucC*_supp_ (*sucC* suppressor 1, which has a SucA Ser_9_STOP mutation) strains were grown aerobically in MHB. The cells were harvested in the exponential phase (6 h), and intracellular metabolites associated with the pyruvate node and TCA cycle were analyzed by LC-MS/MS. The metabolites included in the analysis are indicated in the TCA cycle pathway, along with their associated enzymes. The slit arrow indicates a predicted break in the TCA cycle due to mutation of *sucC* in NE569. *n* = 3; cps, count per second. The image was created using Biorender.com.

Liquid chromatography-tandem mass spectrometry (LC-MS/MS) was used to quantify the accumulation of intracellular metabolites from the TCA cycle and the pyruvate node of glycolysis in NE569 (*sucC*), NE569 p*sucCD*, and *sucC* suppressor strain 1 collected from late-exponential-phase cultures grown aerobically in MHB. Consistent with the predicted impact of a *sucC* mutation, succinyl-CoA levels were significantly increased in NE569 (Fig. 4). Furthermore, accumulation of succinyl-CoA in the *sucC* mutant was accompanied by a concomitant decrease in the levels of succinate (Fig. 4). Succinyl-CoA was reduced to wild-type levels in *sucC* suppressor 1 and the complemented *sucC* mutant, implicating accumulation of this metabolite in *sucC*-dependent modulation of β-lactam resistance. The levels of α-ketoglutarate were also significantly increased in *sucC* suppressor 1 compared to JE2, NE569, and NE569 p*sucCD* (Fig. 4), supporting the conclusion that the SucA Ser_9_STOP mutation in this strain has impaired α-ketoglutarate dehydrogenase activity. Mutation of *sucC* also impacted the glycolytic pathway as evidenced by significantly reduced phosphoenol pyruvate (PEP) and increased levels of pyruvate (Fig. 4). The levels of acetyl-CoA were also significantly reduced in the *sucC* mutant (Fig. 4), as were citrate and isocitrate, albeit not significantly, further demonstrating the impact of this mutation on TCA cycle homeostasis.

### Mutation of *sucC* significantly impacts the global MRSA proteome.

Similar to acetyl-CoA, succinyl-CoA can react with the NH_3_^+^ group located on the lysine side chain of proteins in general resulting in the succinylation of this residue in a pH- and concentration-dependent manner (21, 40). This PTM has been shown to occur extensively in prokaryotes (22, 23, 40–43), and a recent study demonstrated that a murine succinate dehydrogenase mutation altered succinyl-lysine distribution in chromatin (44). Here, the accumulation of succinyl-CoA measured in the *sucC* mutant NE569 prompted us to investigate the global distribution of succinyl-lysines by analyzing the bacterial proteome and succinylome using LC-MS/MS.

For these experiments, cells were collected from JE2 and NE569 cultures grown to exponential and stationary phase as described in Materials and Methods. The global proteome analysis (the workflow is summarized in Fig. S2A in the supplemental material) quantified approximately 90% (*n* = 2,283) of the 2,607 predicted S. aureus proteins. After principal-component analysis and batch correction, JE2 early exponential and exponential-phase samples were found to be largely the same but significantly different from JE2 stationary-phase samples (see Fig. S2B). Compared to JE2, 381 proteins were significantly upregulated in NE569 during exponential growth (see Fig. S2C) and 330 proteins were upregulated in stationary phase (see Fig. S2D), including proteins involved in PG biosynthesis, cell wall organization, and cell division. Significantly downregulated proteins in NE569 during exponential-phase (307 proteins) and stationary-phase (187 proteins) growth included virulence regulators (e.g., SaeS, SaeR, SarR, SarZ, and AgrB), components of type VII secretion systems, hemolysins, leukotoxins, and immune evasion/inactivation proteins (e.g., Spa, Sbi, SraP, and PsmA1) (see Fig. S2C and D), suggesting that virulence of the *sucC* mutant may be attenuated.

10.1128/mBio.00530-21.2 FIG S2 Mutation of *sucC* impacts the MRSA global proteome. Download FIG S2, PDF file, 2.4 MB. Copyright © 2021 Campbell et al. 2021 Campbell et al. https://creativecommons.org/licenses/by/4.0/ This content is distributed under the terms of the Creative Commons Attribution 4.0 International license.

### Global patterns of protein succinylation were increased in the *sucC* mutant.

**FIG 5:**
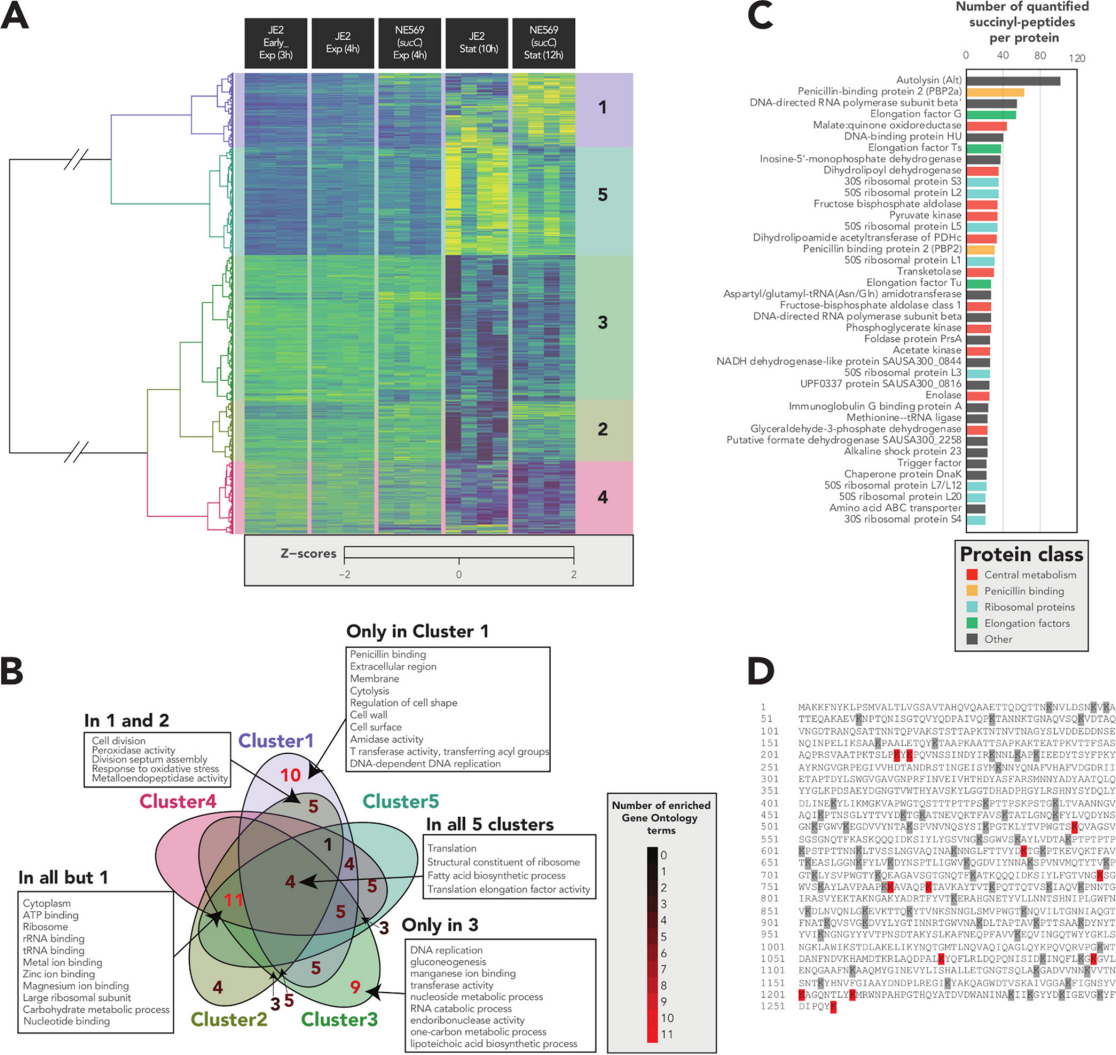
Mutation of *sucC* perturbs lysine succinylation in the S. aureus proteome. (A) Heatmap depicting the quantifiable succinyl-lysine peptides significantly modulated (ANOVA adjusted *P* < 0.05) derived from the proteomes of JE2 and NE569 (*sucC*) collected during early-exponential-, exponential-, and stationary-phase growth. The hierarchical clustering was performed with the Ward method and Euclidean distances. (B) Gene ontology enrichment analysis of succinylated peptides within and shared between heat map clusters. (C) The 40 most succinylated proteins in S. aureus, as indicated by the number of succinylated peptides per protein. (D) Amino acid sequence of Atl highlighting all succinylated lysine residues in gray and lysine residues with significantly increased succinylation in NE569 (*sucC*) versus JE2 in red.

In total 9,545 peptides with succinylated lysine residues (*P* < 0.05, A-Score > 13) (45, 46) were identified. Peptides that could not be quantified in >75% of samples were disregarded, leaving 5,762 succinylated-lysine peptides derived from 1,000 unique proteins. The abundance of approximately 58% of the succinylated peptides (3,340) was significantly modulated across the conditions tested (ANOVA [analysis of variance] adjusted *P* < 0.05) (Fig. 5A). Hierarchical clustering revealed that while most of the changes in the succinylome were growth phase dependent, succinylated peptides in clusters 1 and 2 were more abundant in NE569 compared to JE2 in the stationary phase and were associated with “penicillin binding,” “cytolysis,” “cell wall,” “amidase activity,” “transferase activity transferring acyl-groups,” “response to oxidative stress,” and “metalloendopeptidase activity” (Fig. 5B).

The median number of succinylated peptides per protein was 3, and 670 proteins had <5 succinylated peptides. Intriguingly, the top 40 most succinylated proteins (Fig. 5C) had >20 succinyl peptides each, which represented >20% of all succinylated peptides. Consistent with a recent study in S. epidermidis (23), numerous proteins involved in glycolysis and the TCA cycle were highly succinylated. The major autolysin (Atl) was the most succinylated protein in JE2 and NE569, with the *mecA*-encoded penicillin binding protein 2a (PBP2a) and PBP2 also among the 40 most succinylated proteins (Fig. 5C). Atl had 102 succinylated peptides quantified, mapping to 82 succinyl-lysine sites, of which 12 showed significantly higher levels of succinylation in the *sucC* mutant in the stationary phase (Student *t* test, *P* < 0.05) (Fig. 5D).

### Mutation of *sucC* does not affect *mecA* transcription, PBP2a expression, or peptidoglycan structure and cross-linking.

**FIG 6:**
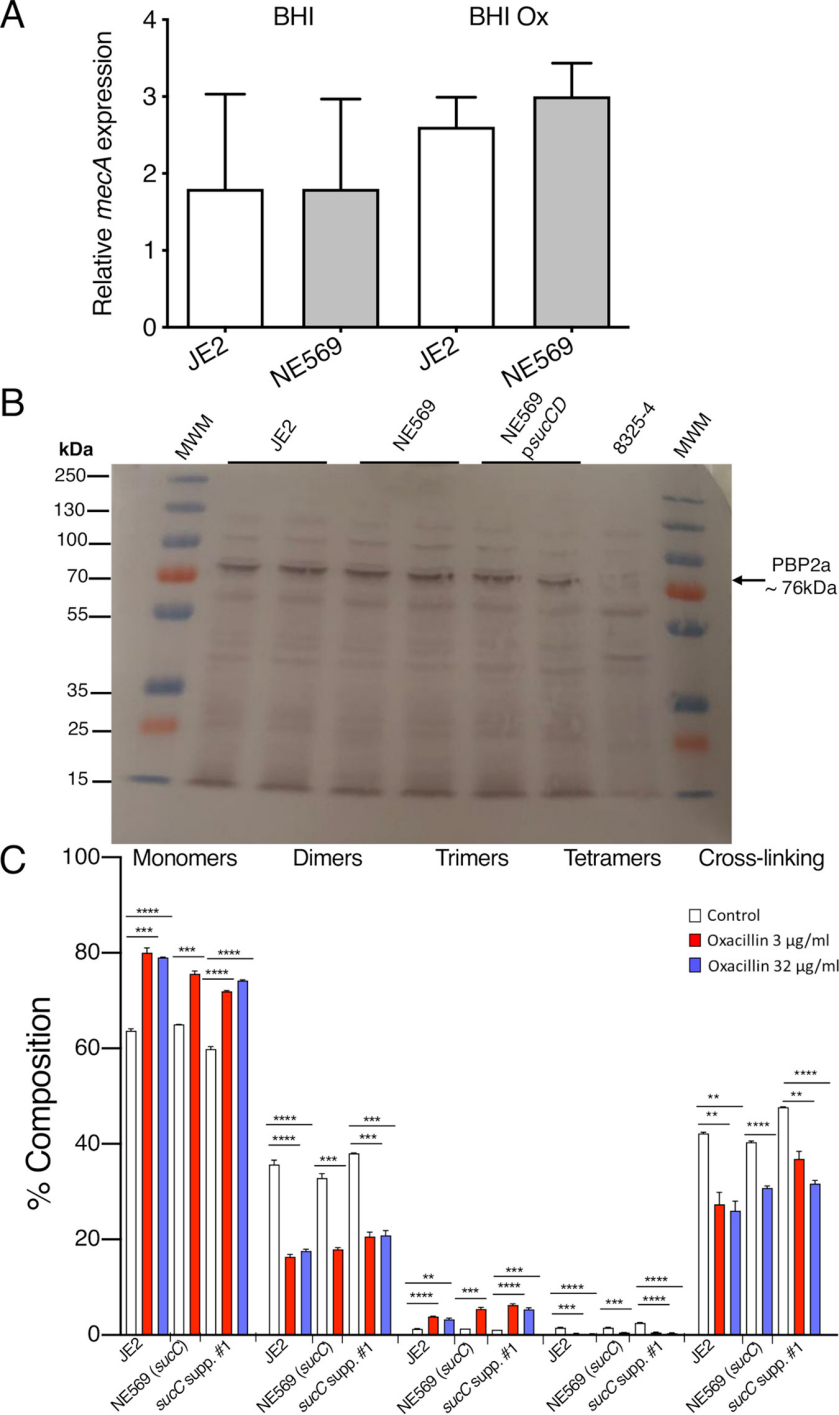
Mutation of *sucC* does not affect *mecA* transcription, PBP2a expression or peptidoglycan structure. (A) Comparison of *mecA* transcription relative to *gyrB* measured by LightCycler RT-qPCR in JE2 and NE569 (*sucC*) grown to exponential phase in BHI or BHI supplemented with 0.5 μg/ml oxacillin. Experiments were repeated at least three times, and standard deviations are shown. Student *t* test analysis revealed no significant differences between either strain or in the presence of absence of oxacillin. (B) Western blotting of PBP2a in JE2, NE569 (*sucC*), NE569 p*sucCD*, and MSSA strain 8325-4 (negative control). Total protein was extracted from cells collected during the exponential phase of growth in MHB plus 2% NaCl supplemented with 0.5 µg/ml oxacillin, with the exception of 8325-4, which was grown without oxacillin. A portion (8 µg) of total protein was separated on a 7.5% Tris-glycine gel, transferred to a polyvinylidene difluoride membrane, and probed with anti-PBP2a antibody (1:1,000 dilution), followed by horseradish peroxidase-conjugated protein G (1:2,000 dilution) and colorimetric detection with a BioRad Opti-4CN substrate kit. Three independent experiments were performed, and a representative blot is shown. (C) Relative proportions of cell wall muropeptide fractions based on oligomerization and relative cross-linking efficiency in peptidoglycan extracted from JE2, NE569 (*sucC*), and *sucC* suppressor 1 grown to exponential phase in MHB or MHB supplemented with oxacillin at 3 or 32 µg/ml. PG analysis from NE569 (*sucC*) is shown only at 3 µg/ml oxacillin because 32 µg/ml exceeds its MIC. Each profile shown is a representative of three biological replicates. Significant differences were determined using a Student *t* test (**, *P* < 0.01; ***, *P* < 0.001; ****, *P* < 0.0001).

LightCycler RT-qPCR analysis revealed that the relative expression of *mecA* was not significantly affected in NE569 compared to JE2 in BHI media or in BHI media supplemented with oxacillin 0.5 μg/ml (Fig. 6A). Western blotting also revealed similar PBP2a levels in JE2, NE569, and NE569 p*sucCD* grown in MHB with 2% NaCl at 35°C supplemented with 0.5 μg/ml oxacillin (Fig. 6B). MSSA strain 8325-4 was included as a *mecA*-negative control.

Quantitative PG compositional analysis was performed using UPLC analysis of muramidase-digested muropeptide fragments extracted from exponential- or stationary-phase cultures of JE2, NE569, and *sucC* suppressor 1 grown in MHB or MHB supplemented with oxacillin 3 μg/ml or 32 μg/ml. The PG profiles of all three strains were similar under all growth conditions tested (see Fig. S3 in the supplemental material). Supplementation of MHB with oxacillin was associated with significant changes in muropeptide oligomerization and reduced cross-linking, but these effects were the same in all strains (Fig. 6C). Interestingly, although PBP2a is the second most succinylated protein in the S. aureus proteome (Fig. 5C) and contains 40 succinylated lysine residues, only the lysine residue K_47_, which is not located near to the enzyme active site or dimerization domains, showed increased succinylation in the *sucC* mutant (MassIVE ID MSV000086976 and MassIVE ID MSV000086971), perhaps making it unlikely that this PTM influences the transpeptidase activity of this enzyme in NE569.

10.1128/mBio.00530-21.3 FIG S3 Mutation of *sucC* does not affect PG structure and crosslinking. Download FIG S3, PDF file, 0.3 MB. Copyright © 2021 Campbell et al. 2021 Campbell et al. https://creativecommons.org/licenses/by/4.0/ This content is distributed under the terms of the Creative Commons Attribution 4.0 International license.

### Mutation of *sucC* does not impact susceptibility to the lipoteichoic acid synthase inhibitor Congo red or the alanylation inhibitor d-cycloserine.

Experiments comparing the susceptibility of JE2 and NE569 to Congo red revealed no differences as evidenced by similar CFU counts, although the morphology of the *sucC* mutant was drastically changed (see Fig. S4). Similarly, the d-cycloserine (DCS) MIC for JE2 (32 μg/ml) was not significantly different from NE569, NE569 p*sucCD*, or *sucC* suppressor 1, all of which had DCS MICs of 16 to 32 μg/ml. Congo red is a selective inhibitor of lipoteichoic acid synthase (LtaS) activity (47), while DCS blocks the d-alanine racemase and d-alanine ligase enzymes required for the production of d-alanine (48, 49), an important precursor for PG, wall teichoic acid (WTA), and lipoteichoic acid (LTA) biosynthesis. Our data already showed that PG structure was unaffected by the *sucC* mutation (Fig. 6C), and these observations further suggest that altered expression or stability of WTA and LTA may not be involved in the reduced β-lactam susceptibility of NE569.

10.1128/mBio.00530-21.4 FIG S4 Mutation of *sucC* does not affect susceptibility to Congo red. Download FIG S4, PDF file, 1.8 MB. Copyright © 2021 Campbell et al. 2021 Campbell et al. https://creativecommons.org/licenses/by/4.0/ This content is distributed under the terms of the Creative Commons Attribution 4.0 International license.

### Autolytic activity is impaired in the *sucC* mutant.

**FIG 7:**
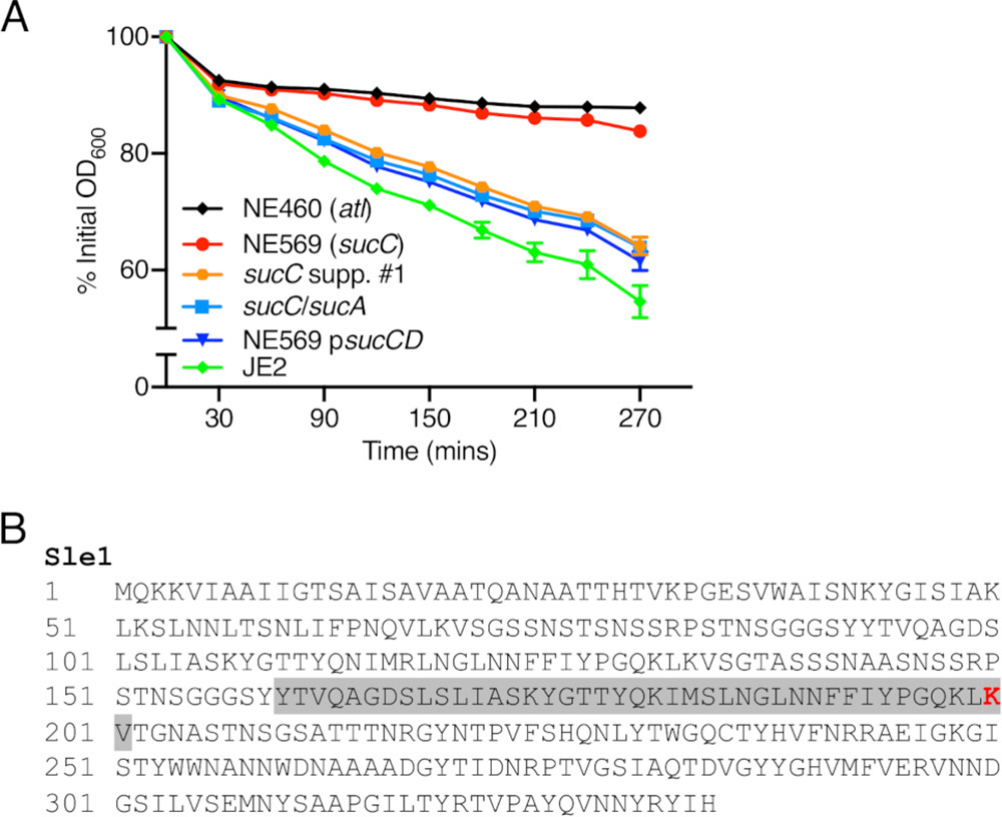
Increased succinylation of lysine residues in Atl and Sle1 is associated with reduced autolytic activity. (A) Triton X-100-induced autolysis of JE2, NE569 (*sucC*), *sucC* suppressor 1, *sucC* *sucA* double mutant, NE569 p*sucCD*, and NE460 (*atl*) strains. The strains were grown to an OD_600_ of 0.5 in MHB at 37°C before being washed in cold PBS and resuspended in 0.1% Triton X-100 with the OD_600_ adjusted to 1. The OD_600_ was monitored at 30-min intervals, and autolysis was expressed as a percentage of the initial OD_600_. The experiments were repeated at least three times, and error bars represent the standard deviations. (B) Amino acid sequence of Sle1 highlighting the K_200_ lysine residue (within a LysM domain [shaded gray]) that is significantly more succinylated in NE569 (*sucC*) versus JE2 in red.

The 12 lysine residues in Atl that exhibited increased levels of succinylation in the *sucC* mutant were evenly distributed throughout the protein, including within the amidase and glucosaminidase domains (Fig. 5D). To investigate whether altered succinylation of Atl impacted its enzymatic activity, Triton X-100-induced autolysis was compared in JE2, NE569, NE569 p*sucCD*, *sucC* suppressor 1, the *sucC sucA* double mutant, and the NE460 (*atl*) mutant. Autolytic activity was strikingly reduced in the *sucC* mutant compared to JE2, and this phenotype was complemented to wild-type levels in NE569 p*sucCD* and reversed in the *sucC*/*sucA* and the *sucA* Ser_9_STOP suppressor mutant strains (Fig. 7). Indeed, Triton X-100-induced autolysis was similar in NE569 and NE460 (Fig. 7). These data suggest that the increased succinylation of the 12 lysine residues in Atl is associated with blocked autolytic activity by interfering with proteolytic cleavage of Atl or the activity of the amidase or glucosaminidase PG hydrolases. Although mutation of *atl* in several MRSA strains, including the HoR strain COL, was previously associated with reduced resistance to methicillin (50), the oxacillin MIC of NE460 (32 μg/ml) was similar to the parent strain JE2. Mutation of Sle1, which like Atl is required for daughter cell separation after cell division, has previously been reported to reduce autolytic activity (51) and increase MRSA susceptibility to oxacillin (52). Succinyl-PTM of the Sle1 K_200_ residue was significantly increased in NE569 compared to JE2 (Student *t* test, *P* < 0.05) (Fig. 7B). Overall, our data reveal that accumulation of succinyl-CoA in the *sucC* mutant negatively impacts two interconnected cell wall-associated phenotypes: autolysis and susceptibility to β-lactam antibiotics.

## DISCUSSION

The TCA cycle is centrally involved in the production of biosynthetic precursors, reducing potential and energy. Here, we report that mutations in *sucC* and *sucD* genes encoding the α and β subunits of succinyl-CoA synthetase, which catalyzes the conversion of succinyl-CoA to succinate, significantly increased susceptibility to β-lactams. The *sucC* and *sucD* mutants grew as smaller, less-pigmented colonies on MHA and exhibited impaired growth in MHB. Genetically blocking the production of succinyl-CoA in the *sucC* mutant by mutating *sucA* or *sucB* reversed the growth and β-lactam susceptibility phenotypes. In contrast, mutation of *sdhA* in the *sucC* mutant had no phenotypic impact. Succinyl-CoA levels were significantly increased in the *sucC* mutant and were restored to wild-type levels by p*sucCD* complementation or mutation of *sucA*.

The accumulation of succinyl-CoA in the *sucC* mutant perturbed global protein succinylation, which is an important PTM previously described in several pathogens (21, 22, 43), including S. epidermidis (23). Although several PBPs, including *mecA*-encoded PBP2a, were among the proteins with the highest number of succinyl-lysines, PG architecture and cross-linking were unchanged in the *sucC* mutant, even under oxacillin stress when *mecA* expression is increased. The absence of structural changes in PG also indicates that the accumulation of succinyl-CoA does not impact β-lactam resistance via altered biosynthesis of lysine, which is an important component of the cell wall (53). Succinyl-CoA is used in the biosynthesis of lysine from aspartate and in Corynebacterium glutamicum disruption of the *sucCD* locus, and the resulting accumulation of succinyl-CoA was accompanied by overproduction of lysine (54). It is difficult to envisage how increased lysine accumulation would reduce β-lactam resistance, and in any event the unchanged PG structure in the *sucC* mutant does not implicate lysine biosynthesis in this phenotype. Succinyl-CoA synthetase activity generates GTP, which is a substrate for RelA, RelP, and RelQ enzymes that produce the stringent response alarmone (p)ppGpp. (p)ppGpp plays a central role in the control of MRSA β-lactam resistance (31–34). However, the ability of the *sucC* mutant to produce stable HoRs with mutations in *relA* or *relQ* suggests that intracellular GTP is not limited in the *sucC* and *sucD* mutants or associated with reduced β-lactam resistance. Metabolomic analysis further revealed significantly reduced levels of acetyl-CoA in the *sucC* mutant, raising the additional possibility that the acetylome may also have been perturbed. In this context, changes in PG acetylation have also been implicated in autolysis and antibiotic resistance (55), and future comparison of the acetylome and succinylome in *sucCD* mutants to identify proteins that are modified by both PTMs may provide further insights into how these mutations impact resistance.

The reduced pigmentation of the *sucCD* mutants may be a consequence of altered production of the S. aureus carotenoid staphyloxanthin, composed of a glucose residue esterified with a 30-carbon carboxylic acid chain and a 15-carbon fatty acid (56). In S. aureus most fatty acids are odd-numbered branched-chain fatty acids (57). The β-oxidation of odd-numbered fatty acids generates acetyl-CoA and propionyl-CoA, the latter of which can be converted to succinyl-CoA. Interestingly, staphyloxanthin-derived lipids interact with flotillin to form functional membrane microdomains required for oligomerization and activity of PBP2a (58). In contrast to the reduced pigmentation, the proteomic analysis revealed increased levels of staphyloxanthin biosynthetic enzymes in the *sucC* mutant (MassIVE ID MSV000086976 and MassIVE ID MSV000086971), perhaps reflecting efforts by the *sucC* mutant to compensate for reduced pigmentation.

The discovery that Atl was the most succinylated protein in the MRSA proteome and that 12 of the 82 succinyl-lysines were significantly more succinylated in the *sucC* mutant suggested a potential connection to reduced β-lactam resistance. Fisher and Mobashery recently proposed a model in which the bactericidal activity of β-lactams is the result of deregulated Atl activity at the cell division septum (59). Our data showing that autolytic activity was significantly reduced in the *sucC* mutant and that the oxacillin MIC of the *atl* transposon mutant NE460 was unchanged are not consistent with this possibility. Furthermore, previous work in our laboratory linked increased autolytic activity with increased β-lactam resistance. Specifically, *atl* transcription was activated in a HoR mutant of USA300 LAC (oxacillin MIC > 256 µg/ml), which exhibited significantly increased autolytic activity, and growth of USA300 LAC in sub-MIC oxacillin was also associated with significantly increased autolysis (60). Thus, while it seems clear that autolysis and β-lactam susceptibility are interconnected, the precise mechanistic interactions between these two phenotypes needs to be elucidated further.

Processing of the Atl proprotein, produces a signal peptide, a propeptide, a *N*-acetylmuramoyl-l-alanine amidase (AM) enzyme, and a C-terminally located endo-β-*N*-acetylglucosaminidase enzyme (GL) (61). The region between the AM and GL catalytic domains contains three repeat regions (R1 to R3) with GW-dipeptide motifs required to target Atl proprotein to the equatorial ring on the cell surface during cell division (62). None of these 12 lysine residues exhibiting increased succinylation are in the AM catalytic domain, 5 are in the GL catalytic domain, 2 are in the propeptide region, and the remaining 5 are in the R1 and R2 regions. The single lysine residue (K_200_) of Sle1 that is more succinylated in the *sucC* mutant is located in a LysM cell wall hydrolase domain and may be important for activity of the enzyme.

**FIG 8:**
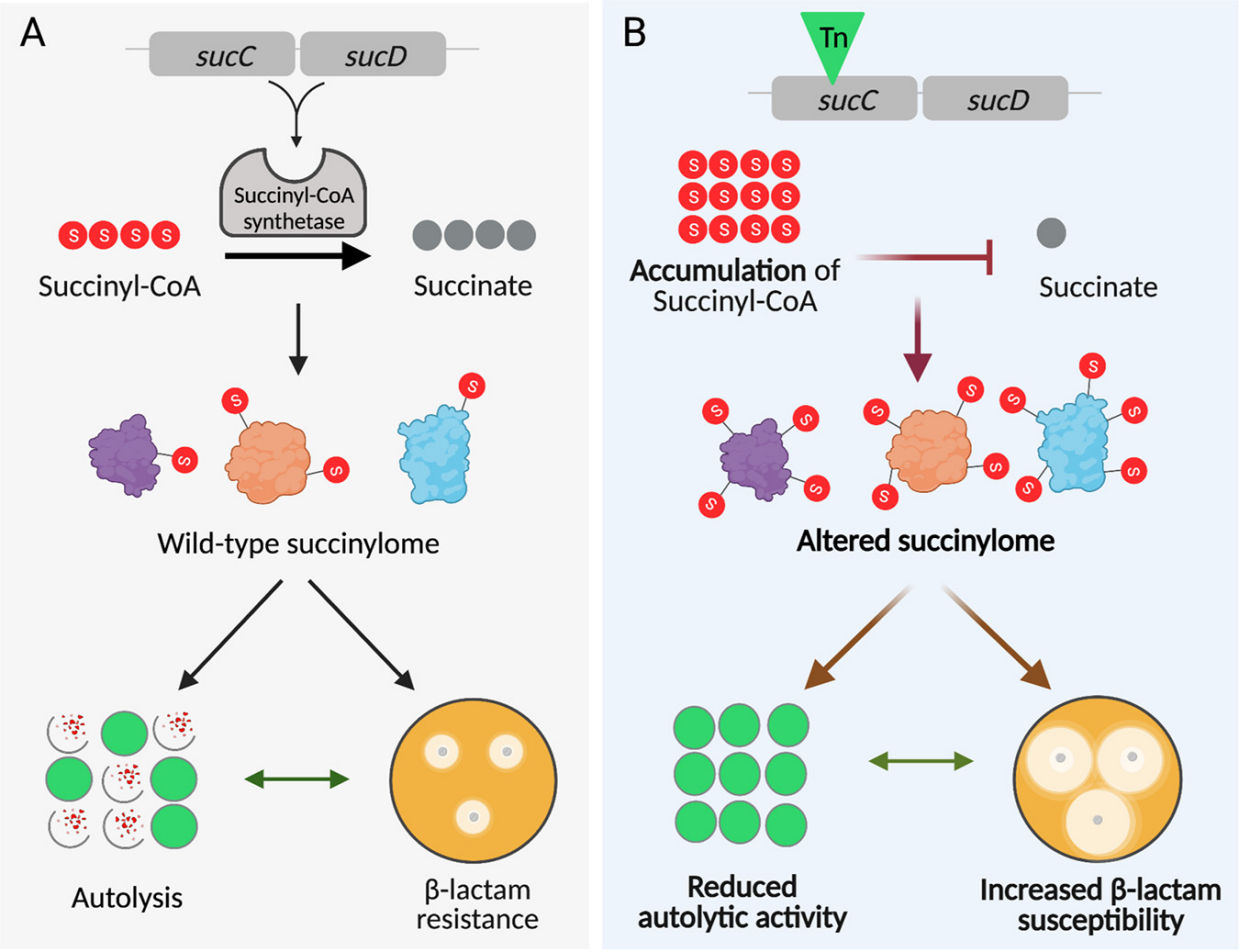
Suggested model for succinylome-controlled regulation of two interconnected cell wall-associated phenotypes, namely, autolysis and β-lactam susceptibility, in MRSA. Image created with Biorender.com.

Susceptibility to the LtaS inhibitor Congo red was unchanged in the *sucC* mutant, indicating that increased β-lactam susceptibility may not be associated with impaired expression or stability of WTA or LTA. Similarly, susceptibility to the alanylation inhibitor DCS was the same in the wild-type and *sucC* mutant. Given that d-alanine is an important component of PG, this observation is consistent with the absence of any changes in PG structure in the *sucC* mutant. Furthermore, because d-alanine is an important component of WTA and LTA, unchanged susceptibility to DCS does not point to roles for these cell envelope glycopolymers in *sucC*-dependent β-lactam susceptibility. Nevertheless, given that almost 58% of all quantifiable succinylated peptides were significantly changed in the *sucC* mutant, we propose a model in which perturbation of the succinylome likely modulates the activity of multiple enzymes, including Atl and Sle1, that collectively control growth and interconnected cell envelope characteristics such as autolysis and susceptibility to β-lactam antibiotics (Fig. 8). The U.S. Food and Drug Administration-approved anticancer drug streptozotocin specifically targets succinyl-CoA synthetase in human cells to limit proliferation (63) and is used primarily to treat tumors that cannot be surgically removed. The findings described here may open the door to the possibility of sensitizing MRSA to β-lactam antibiotics using compounds that specifically target succinyl-CoA synthetase or protein succinylation generally within the cell.

## MATERIALS AND METHODS

### Bacterial strains and growth conditions.

Bacterial strains and plasmids used in this study are listed in Table S1 in the supplemental material. Escherichia coli strains were cultured in Luria-Bertani (LB) broth or LB agar. S. aureus strains were grown in Mueller-Hinton broth (MHB), Mueller-Hinton agar (MHA), tryptic soy broth (TSB), brain heart infusion (BHI), tryptic soy agar (TSA), chemically defined medium (CDM), and CDM supplemented with glucose (CDMG) and, where indicated, supplemented with erythromycin (Erm) at 10 µg/ml, chloramphenicol (Cm) at 10 µg/ml, ampicillin (Amp) at 50 µg/ml, or kanamycin (Km) at 75 µg/ml. For growth experiments in MHB, 25 or 50 ml of MHB in 250-ml flasks were used for a 10:1 or 5:1 flask/volume ratio, respectively. Overnight cultures in MHB were used to inoculate the media at a starting optical density at 600 nm (OD_600_) of 0.01, and flasks were incubated at 37°C shaking at 200 rpm. For CDM and CDMG growth experiments, overnight cultures were grown in TSB, washed once in 5 ml of phosphate-buffered saline (PBS), and used to inoculate 25 ml of CDMG or CDM in a 250-ml flask to a starting OD_600_ of 0.05. Flasks were incubated at 37°C with shaking at 200 rpm. For all growth experiments, CFU were enumerated in serially diluted 20-µl aliquots removed from flask cultures. At least three biological replicates were performed for each strain, and average data are presented. For experiments to compare growth in MH and in MH supplemented with 5% glucose, cultures were grown at 37°C in 250-ml flasks with 50 ml of media and shaking at 200 rpm.

10.1128/mBio.00530-21.5 TABLE S1 Bacterial strains and plasmids used in this study. Download Table S1, DOCX file, 0.03 MB. Copyright © 2021 Campbell et al. 2021 Campbell et al. https://creativecommons.org/licenses/by/4.0/ This content is distributed under the terms of the Creative Commons Attribution 4.0 International license.

### Cefoxitin disk diffusion assays and MIC measurements.

Cefoxitin disk diffusion susceptibility testing was performed in accordance with Clinical and Laboratory Standards Institute (CLSI) guidelines (64). MIC measurements by broth microdilution or agar dilution were performed in accordance with CLSI methods for dilution susceptibility testing of staphylococci (65). Disk diffusion and MIC results were interpreted using CLSI standard M100, and strains were classified as susceptible or resistant (66). For oxacillin MIC measurement, M.I.C.Evaluator (Oxoid) strips were used in accordance with manufacturer guidelines. Strains were grown at 37°C on MHA for 24 h, 5 to 6 colonies were resuspended in 0.85% saline and adjusted to a 0.5 McFarland standard. The suspension was swabbed evenly three times across the surface of an MHA 2% NaCl plate (4-mm agar depth). An M.I.C.Evaluator strip was applied, followed by incubation for 24 h at 35°C. Three independent measurements were performed for each strain.

### Genomic DNA extraction and whole-genome sequencing.

Genomic DNA extraction, sequencing by MicrobesNG (http://www.microbesng.uk), and analysis were performed as described previously (67). Wild-type JE2 reads were aligned against the published USA300 FPR3757 genome sequence (RefSeq accession number NC_007793.1) and assembled into a contig. USA300 FPR3757 annotation and base numbering were transferred onto the wild-type JE2 sequence. The reads for *sucC* suppressor strains and *sucC* HoR strains were then mapped onto the assembled JE2 sequence. High-frequency (70%) and good-quality base changes were identified using the CLC Genomics Workbench software package (version 20.0.2). Sequence data for NE569 (*sucC*), *sucC* HoR1, *sucC* HoR2, and *sucC* suppressors 1, 2, and 3 are available from the European Nucleotide Archive (project PRJEB43960, accession numbers ERS6142066 to ERS6142071).

### Genetic manipulation of *S. aureus*.

Phage 80α was used to transduce the transposon insertion from NE569 into JE2, DAR173, and USA300 to ensure that background mutations were not responsible for the antibiotic resistance phenotype, as described previously (67). Transposon insertions were verified using PCR amplification of target loci.

A 1,680-bp fragment encompassing the *sucC* gene and a 2,610-bp fragment, including both *sucC* and *sucD*, were PCR amplified from JE2 genomic DNA using the primers INF_sucC_F/INF_sucC_R and INF_sucCD_F/INF_sucCD_R, respectively (see Table S2 in the supplemental material) and cloned into the E. coli-Staphylococcus shuttle vector, pLI50, using Clontech infusion cloning kit 2. The p*sucCD* plasmid was digested with HpaI and SpeI and treated with T4 DNA polymerase (Roche) and deoxynucleoside triphosphates to create a blunt-end fragment that was religated using T4 ligase (Roche). The resulting plasmid, designated p*sucD*, contained a 627-bp deletion at the 5′ end of the *sucC* gene, with only the *sucD* gene remaining intact. Recombinant plasmids were first transformed into cold-competent E. coli HST08 (supplied with the Clontech infusion cloning kit) before being transformed by electroporation into the restriction-deficient S. aureus strain RN4220 and finally into NE569 and NE1770.

10.1128/mBio.00530-21.6 TABLE S2 Oligonucleotide primers used in this study. Download Table S2, DOCX file, 0.02 MB. Copyright © 2021 Campbell et al. 2021 Campbell et al. https://creativecommons.org/licenses/by/4.0/ This content is distributed under the terms of the Creative Commons Attribution 4.0 International license.

The *sucC sucA*, *sucC sdhA*, and *sucC relA* double mutants were generated by first exchanging the *sucC*::Tn containing an Erm^r^ cassette in NE569 for a transposon containing a Kan^r^ cassette, generating strain *sucC*::Tn-Kan^r^. This was performed by allelic exchange, using the plasmid pKAN and a method described previously (68). Genomic DNA extraction and PCR using the primers suc_F and sucC_R was carried out to verify the allelic exchange process.

To construct the *sucC relA* double mutant, the *sucC*::Tn-Kan^r^ was transduced into NE1714 and transductants were selected on TSA with 75 µg/ml kanamycin. To construct *sucC* *sucA* and *sucC* *sdhA* double mutants, the *sucA*::Tn allele from NE547 and the *sdhA*::Tn allele of NE626 were each transduced into *sucC*::Tn-Kan^r^ using phage 80α, and transductants were selected on TSA with 10 µg/ml erythromycin. PCR was used to verify the transposon insertions in the double mutants using primers for *sucC*, *relA*, *sucA*, and *sdhA* (Table S2).

### RNA purification and real-time RT-PCR.

Cultures were grown in BHI media to midexponential phase. Harvested cells were pelleted and immediately stored at –20°C in RNAlater (Ambion) to ensure maintenance of RNA integrity. RNA was extracted according to the manufacturer’s guidelines using an RNA Mini-Extraction kit (Sigma). RNA integrity was examined visually by agarose gel electrophoresis and RNA concentration was determined using a Qubit Fluorometer 4 (Qiagen). Quantitative reverse transcription-PCR (RT-qPCR) was used to measure *mecA* transcription on the Roche LightCycler 480 instrument using a LightCycler 480 SYBR green kit (Roche) with the primers mecA1_Fwd and mecA1_Rev (see Table S2), as described previously (49). The *gyrB* gene amplified with the primers gyrB_Fwd and gyrB_Rev (see Table S2) served as an internal standard. Each RT-qPCR experiment was performed three times, and average data are presented, including standard deviations.

### PBP2a Western blot analysis.

Overnight MHB cultures were used to inoculate 25 ml of MHB plus 2% NaCl, with or without 0.5 µg/ml oxacillin, to a starting OD_600_ of 0.05, followed by incubation at 35°C (with 200-rpm shaking) until an OD_600_ of 0.8 was reached. The cells were then pelleted and resuspended in PBS to an OD_600_ of 10, and PBP2a Western blot analyses were performed as described previously (67). Three independent experiments were performed and a representative blot presented.

### Isolation of *sucC* suppressor mutants.

For experiments comparing the growth of JE2 and the *sucC* mutant NE569, aliquots removed from 25-ml MHB cultures grown in 250-ml flasks were serially diluted, and the CFU were enumerated on MHA. Suppressor mutants of NE569 were readily identified on MHA due to their larger colony size and deeper pigmentation, which contrasted with the smaller, pale colony morphology of parental NE569. Three suppressor mutants (1, 2, and 3) were chosen for further analysis. DNA extraction and PCR verification of the *Bursa aurealis* transposon using the primers sucC_F and sucC_R (see Table S2) was performed to verify the presence of the *sucC* transposon insertion. Comparative genome sequence analysis was used to identify the suppressor mutations.

### Oxacillin resistance population analysis.

Population analysis profiles (PAPs) was generated as described previously (69). Overnight cultures were grown in BHI, adjusted to an OD_600_ of 1, and 10-fold serially diluted from 10^−1^ to 10^−7^, and a 20 µl aliquot of each dilution was plated onto a series of BHI agar plates supplemented with oxacillin at 0.25, 0.5, 1, 2, 4, 8, 16, 32, 64, or 128 µg/ml. The CFU were enumerated after overnight incubation at 37°C, and the results were expressed as the CFU/ml at each oxacillin concentration. Average data from three independent experiments are presented.

### Isolation of homogeneously resistant mutants.

Overnight cultures of NE569 were grown in BHI, adjusted to an OD_600_ of 1, serially diluted, plated onto BHI agar supplemented with 100 µg/ml oxacillin, and incubated at 37°C to isolate HoR mutants. The CFU were enumerated on BHI agar to calculate the rate of HoR mutant production. HoR mutants were passaged for 14 days in antibiotic-free BHI broth to identify stable mutants that were then verified using PAPs and oxacillin MIC measurements. The experiments were performed twice, and the results of a representative experiment are presented.

### LC-MS/MS metabolite analysis of NE569 (*sucC*), NE569 p*sucCD*, and *sucC* suppressor 1.

For LC-MS/MS analysis, samples were prepared as previously described (70). Briefly, strains were inoculated in MHB to an OD_600_ of 0.06 and grown aerobically (250 rpm, 37°C) for 6 h. Culture volumes corresponding to OD_600_ of 10 were harvested and rapidly filtered through a membrane (0.45 μm; Millipore). The cells on the membrane were washed twice with 5 ml of cold saline and immediately quenched in ice-cold 60% ethanol containing 2 μM Br-ATP as an internal control. The cells were mechanically disrupted using a bead homogenizer set to oscillate for three cycles (30 s) of 6,800 rpm with a 10-s pause between each cycle. Cell debris was separated by centrifugation at 13,000 rpm. The supernatant containing intracellular metabolites were lyophilized and then stored at −80°C.

A triple-quadrupole-ion trap hybrid mass spectrometry (Sciex) connected with a Waters ultraperformance liquid chromatography I-class (UPLC) system was used for the metabolite analysis. The chromatographic separation was performed by ionic liquid chromatography using a Column XSELECT HSS XP (150 mm × 2.1 mm inner diameter; 2.5-μm particle size), and a binary solvent system with a flow rate of 0.250 ml/min was used for chromatographic separation. Mobile phase A was composed of 10 mM tributylamine, 10 mM acetic acid, 5% methanol, and 2% 2-propanol; mobile phase B was 100% methanol. The column was maintained at 40°C, and the autosampler was maintained at 7°C. The A/B solvent ratio was maintained at 90/10 for 1 min, followed by a gradual increase in B to 65% for 10 min. Solvent B was increased to 90% over next 1 min, which was maintained for 4 min. The gradient was again reduced to 90/10 (A/B) within 0.5 min and was equilibrated for 5.5 min before the next run. A QTRAP 6500+ mass spectrometry system (Sciex) operated in negative-ion mode was used for targeted quantitation in multiple reaction monitoring (MRM) mode. MRM details for each analyte are listed in Table S3 in the supplemental material. The electrospray ionization parameters were optimized for a 0.25-ml/min flow rate and were as follows: an electrospray ion voltage of −4,400 V, a source temperature of 400°C, a curtain gas of 40, and gas 1 and 2 of 40 and 45 lb/in^2^, respectively. Analyzer parameters were optimized for each compound using manual tuning.

10.1128/mBio.00530-21.7 TABLE S3 MRM transitions for all metabolites measured in this study. Download Table S3, DOCX file, 0.01 MB. Copyright © 2021 Campbell et al. 2021 Campbell et al. https://creativecommons.org/licenses/by/4.0/ This content is distributed under the terms of the Creative Commons Attribution 4.0 International license.

### Proteomics sample preparation and analysis.

Cells were collected from four biological replicates of JE2 and NE569 (*sucC*) flask cultures grown to exponential and stationary phases in MHB. Due to different growth characteristics and CFU/ml in wild-type and *sucC* mutant cultures (see Fig. S1), JE2 and NE569 cells were collected after 3 and 4 h, respectively, for exponential growth and after 10 and 12 h, respectively, for stationary-phase growth. As an added control four biological replicates of JE2 cells were also collected after 3 h (early exponential phase). Cell pellets were collected promptly from culture samples and snap-frozen in liquid nitrogen.

The cell pellets from all 20 samples were lysed, denatured, and digested with trypsin before being labeled with tandem mass tags (TMT) using TMT11plex, as described previously (71). Because there were 20 samples and only 11 channels in the TMT11plex kit, an equal concentration of peptides from all the cell pellets was mixed to generate a reference sample used for normalization purposes. Two samples from each cell pellet were labeled with different isobaric labels and placed in each of the two TMT sets, the channel 131C was reserved for the reference sample. The peptide samples were cleaned-up by C_18_ solid-phase extraction (SPE). An immunoaffinity purification was performed using the PTMScan acetyl-lysine motif (Ac-K) kit (Cell Signaling Technology, Danvers, MA) to bind acetyl peptides, thereby enriching succinyl-lysine peptides in the unbound fraction, which was cleaned-up using a C_18_ SPE and fractionated (into 12 fractions) using high-pH reverse-phase chromatography as described previously (72). Peptides were analyzed by reverse-phase separation (C_1_8) coupled with a QExactive HF-X mass spectrometer. The instrument .raw files generated were deposited and are available at MassIVE data repository (MassIVE ID MSV000086976 and MassIVE ID MSV000086971). Raw MS data were searched with MS-GF+ (73) against UniProt/Swiss-Prot S. aureus database (UP000001939) bovine trypsin and human keratin sequences. Methionine oxidation and succinylation were set as dynamic modifications, and cysteine alkylation and TMT labeling of N termini and lysines were set as static modifications. The identified spectra were filtered based on their MS-GF+ scores, resulting in a false discovery rate of <1%. For quantitative analysis, the TMT reporter ion intensities were extracted with MASIC (74). Intensities were normalized to reference channel intensity. Analytes were considered for quantification when reporters were observed for at least three-quarters of the samples per given condition. The subsequent data were processed using the package RomicsProcessor (http://doi.org/10.5281/zenodo.3956544). Briefly, the data were log_2_ transformed, median centered, and batch corrected using the sva ComBat method (75), before missing values were imputed using random values drawn from a normal distribution downshifted by 2 standard deviations with a width of 0.5 standard deviation similar to the Perseus imputation method (76). ANOVA and Student *t* tests were used to determine significance. The DAVID modified Fisher exact test (EASE score) (77) was used to measure significant enrichment of Gene Ontology (GO) terms in proteins whose abundance was altered by the *sucC* mutation.

### Peptidoglycan analysis.

Wild-type JE2, NE569, and *sucC* suppressor 1 were grown in MHB and in MHB supplemented with oxacillin at 3 or 32 μg/ml. For each strain and growth condition tested, independent quadruplicate 50-ml cultures were grown in flasks at 37°C with 200 rpm shaking to an OD_600_ of 0.5, and cell pellets were collected promptly and snap-frozen in liquid nitrogen. PG was extracted from the samples as described previously (49, 78). This analysis of wild-type JE2 PG was also used as a part of a separate study (67) and is reported again here for comparison to NE569 and *sucC* suppressor 1. Mass spectrometry (MS) was performed on a Waters XevoG2-XS QTof mass spectrometer. Structural characterization of muropeptides was determined based on their MS data and MS/MS fragmentation pattern, matched with the PG composition and structure reported previously (79–82).

### Autolytic activity assays.

Samples (200 μl) from overnight cultures were inoculated into 20 ml of TSB, grown at 37°C (200 rpm) to an OD_600_ of 0.5, washed with 20 ml of cold PBS, resuspended in 5 ml of cold PBS, and then adjusted to an OD_600_ of 1. Then, 1 ml of the cell suspension was transferred to a cuvette, and Triton X-100 was added at a final concentration of 0.1% (vol/vol). The initial OD_600_ was recorded before incubation at 37°C with shaking (200 rpm). Thereafter, the OD_600_ was recorded every 30 min for 4 h, and autolytic activity is expressed as a percentage of the initial OD_600_. NE460 (*atl*::Tn) was used as a control, and at least three biological replicates were performed for each strain.

### Data availability.

Proteomic .raw files generated during this study are available at MassIVE data repository (MassIVE ID MSV000086976 and MassIVE ID MSV000086971). The UniProt/Swiss-Prot S. aureus database (UP000001939) was used as a reference for the proteomic analysis.

Whole-genome sequence data are available from the European Nucleotide Archive (project PRJEB43960, accession numbers ERS6142066 to ERS6142071). The SAUSA300_FRP3757 (TaxID:451515) reference genome sequence is available from the National Center for Biotechnology Information.
