# Supplementary material for: Accumulation of Succinyl Coenzyme A Perturbs the Methicillin-Resistant Staphylococcus aureus (MRSA) Succinylome and Is Associated with Increased Susceptibility to Beta-Lactam Antibiotics
Source: mBio. 2021 Jun 29;12(3):e00530-21. doi: 10.1128/mBio.00530-21 (PMC8437408; doi:10.1128/mBio.00530-21)
Supplement: FIG S1 [file mbio.00530-21-sf001.pdf]

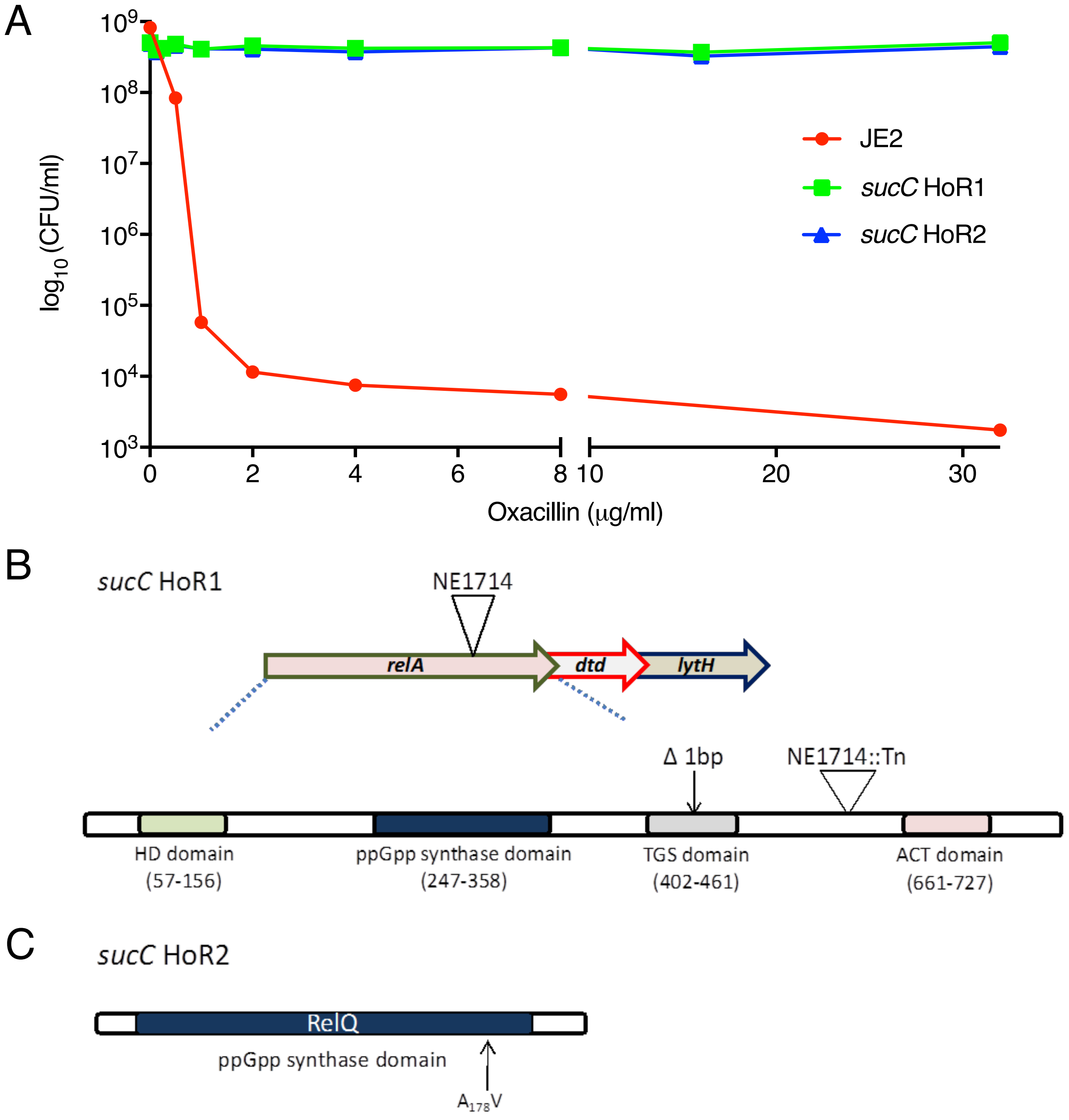

Fig. S1. NE569 (*sucC*) HoR mutants contain RSH (*relA*) and *relQ* mutations. A. Population analysis profiling of JE2, *sucC* HoR1 and *sucC* HoR2 grown in BHI broth to exponential phase, adjusted to OD_{600} of 1 and serially diluted before plating onto BHI agar supplemented with increasing concentrations of oxacillin (0-100 mg/ml) for enumeration of CFUs. This assay was repeated three times and data from a representative experiment is shown. **B.** *relA-dtd-lytH* operon in *S. aureus* and RSH (RelA) domain architecture including the location of the 1bp deletion in *sucC* HoR1 and the Tn insertion in NE1714. **C.** Location of A178V mutation in ppGpp synthase domain of RelQ in strain sucC HoR2.
