# Supplementary material for: Accumulation of Succinyl Coenzyme A Perturbs the Methicillin-Resistant Staphylococcus aureus (MRSA) Succinylome and Is Associated with Increased Susceptibility to Beta-Lactam Antibiotics
Source: mBio. 2021 Jun 29;12(3):e00530-21. doi: 10.1128/mBio.00530-21 (PMC8437408; doi:10.1128/mBio.00530-21)
Supplement: FIG S2 [file mbio.00530-21-sf002.pdf]

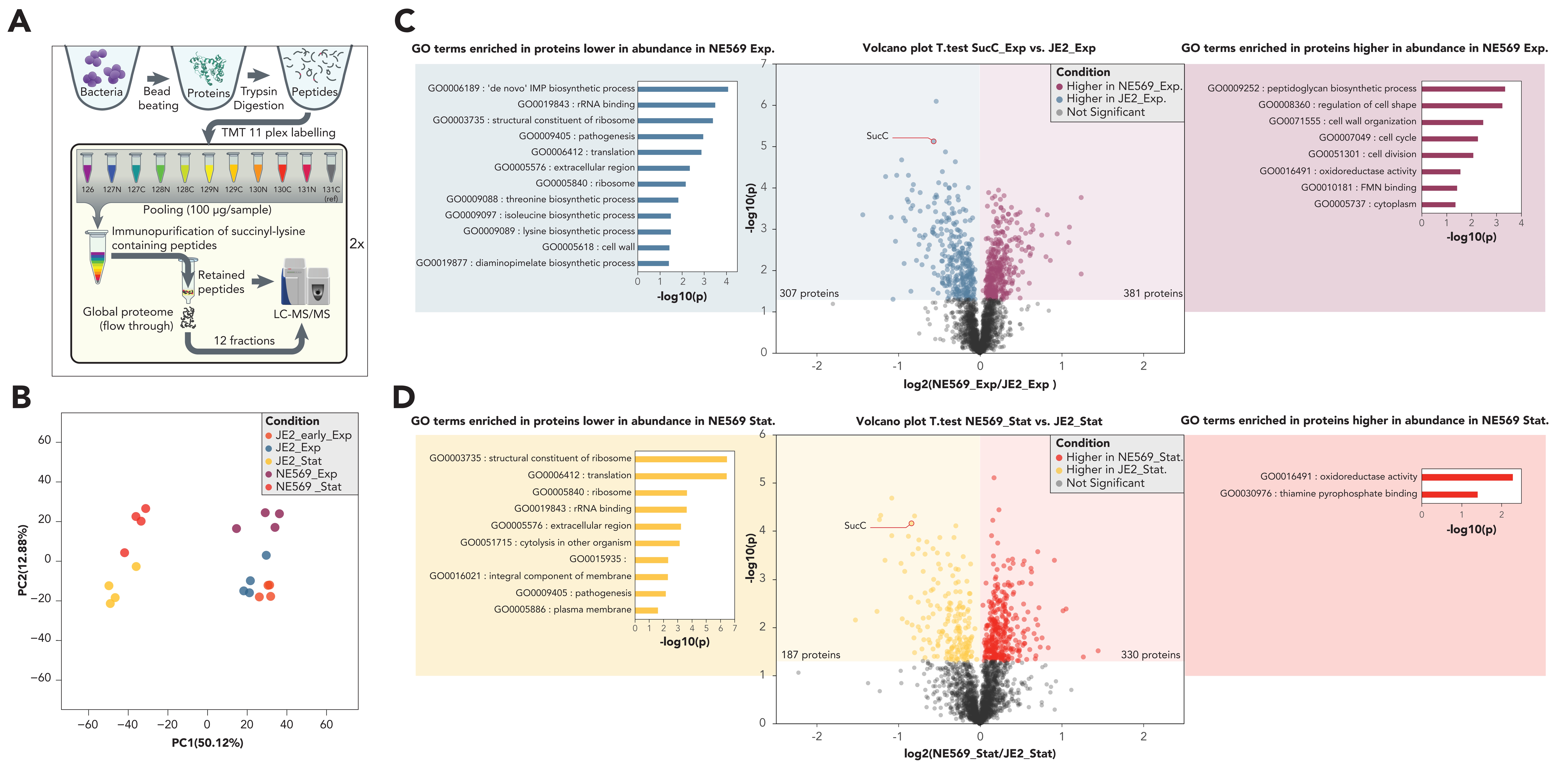

A
Bacteria
Bead beating
Proteins
Trypsin Digestion
Peptides
TMT 11 plex labelling
126 127N 127C 128N 128C 129N 129C 130N 130C 131N 131C (ref)
Pooling (100 µg/sample)
Immunopurification of succinyl-lysine containing peptides
2x
Retained peptides
Global proteome (flow through)
LC-MS/MS
12 fractions
B
Condition
JE2_early_Exp
JE2_Exp
JE2_Stat
NE569_Exp
NE569 _Stat
PC2(12.88%)
PC1(50.12%)
C
GO terms enriched in proteins lower in abundance in NE569 Exp.
GO0006189 : 'de novo' IMP biosynthetic process
GO0019843 : rRNA binding
GO0003735 : structural constituent of ribosome
GO0009405 : pathogenesis
GO0006412 : translation
GO0005576 : extracellular region
GO0005840 : ribosome
GO0009088 : threonine biosynthetic process
GO0009097 : isoleucine biosynthetic process
GO0009089 : lysine biosynthetic process
GO0005618 : cell wall
GO0019877 : diaminopimelate biosynthetic process
-log10(p)
Volcano plot T.test SucC_Exp vs. JE2_Exp
Condition
Higher in NE569_Exp.
Higher in JE2_Exp.
Not Significant
SucC
307 proteins
381 proteins
-log10(p)
log2(NE569_Exp/JE2_Exp)
GO terms enriched in proteins higher in abundance in NE569 Exp.
GO0009252 : peptidoglycan biosynthetic process
GO0008360 : regulation of cell shape
GO0071555 : cell wall organization
GO0007049 : cell cycle
GO0051301 : cell division
GO0016491 : oxidoreductase activity
GO0010181 : FMN binding
GO0005737 : cytoplasm
-log10(p)
D
GO terms enriched in proteins lower in abundance in NE569 Stat.
GO0003735 : structural constituent of ribosome
GO0006412 : translation
GO0005840 : ribosome
GO0019843 : rRNA binding
GO0005576 : extracellular region
GO0051715 : cytolysis in other organism
GO0015935 :
GO0016021 : integral component of membrane
GO0009405 : pathogenesis
GO0005886 : plasma membrane
-log10(p)
Volcano plot T.test NE569_Stat vs. JE2_Stat
Condition
Higher in NE569_Stat.
Higher in JE2_Stat.
Not Significant
SucC
187 proteins
330 proteins
-log10(p)
log2(NE569_Stat/JE2_Stat)
GO terms enriched in proteins higher in abundance in NE569 Stat.
GO0016491 : oxidoreductase activity
GO0030976 : thiamine pyrophosphate binding
-log10(p)

Fig. S2. Mutation of *sucC* impacts the MRSA global proteome. A. Workflow for MRSA proteome and succinylome analysis by LC-MS/MS. **B.** Principal component analysis grouping protein samples by strain and growth phase. **C.** Comparison of the relative abundance of proteins from JE2 and NE569 (*sucC*) exponential phase cultures. The volcano plot depicts the fold-change and the -log10(p) of the Student's t-test. The bar plots show the GO terms significantly enriched (EASE modified Fisher's exact $p<0.05$) either for the protein significantly less abundant in NE569 (left) or more abundant in NE569 (right). **D.** Comparison of the relative abundance of proteins from JE2 and NE569 (*sucC*) stationary phase cultures. The volcano plot depicts the fold-change and the -log10(p) of the Student's t-test. The bar plots show the GO terms significantly enriched (EASE modified Fisher's exact $p<0.05$) either for the protein significantly less abundant in NE569 (left) or more abundant in NE569 (right).
