# Supplementary material for: Accumulation of Succinyl Coenzyme A Perturbs the Methicillin-Resistant Staphylococcus aureus (MRSA) Succinylome and Is Associated with Increased Susceptibility to Beta-Lactam Antibiotics
Source: mBio. 2021 Jun 29;12(3):e00530-21. doi: 10.1128/mBio.00530-21 (PMC8437408; doi:10.1128/mBio.00530-21)
Supplement: FIG S3 [file mbio.00530-21-sf003.pdf]

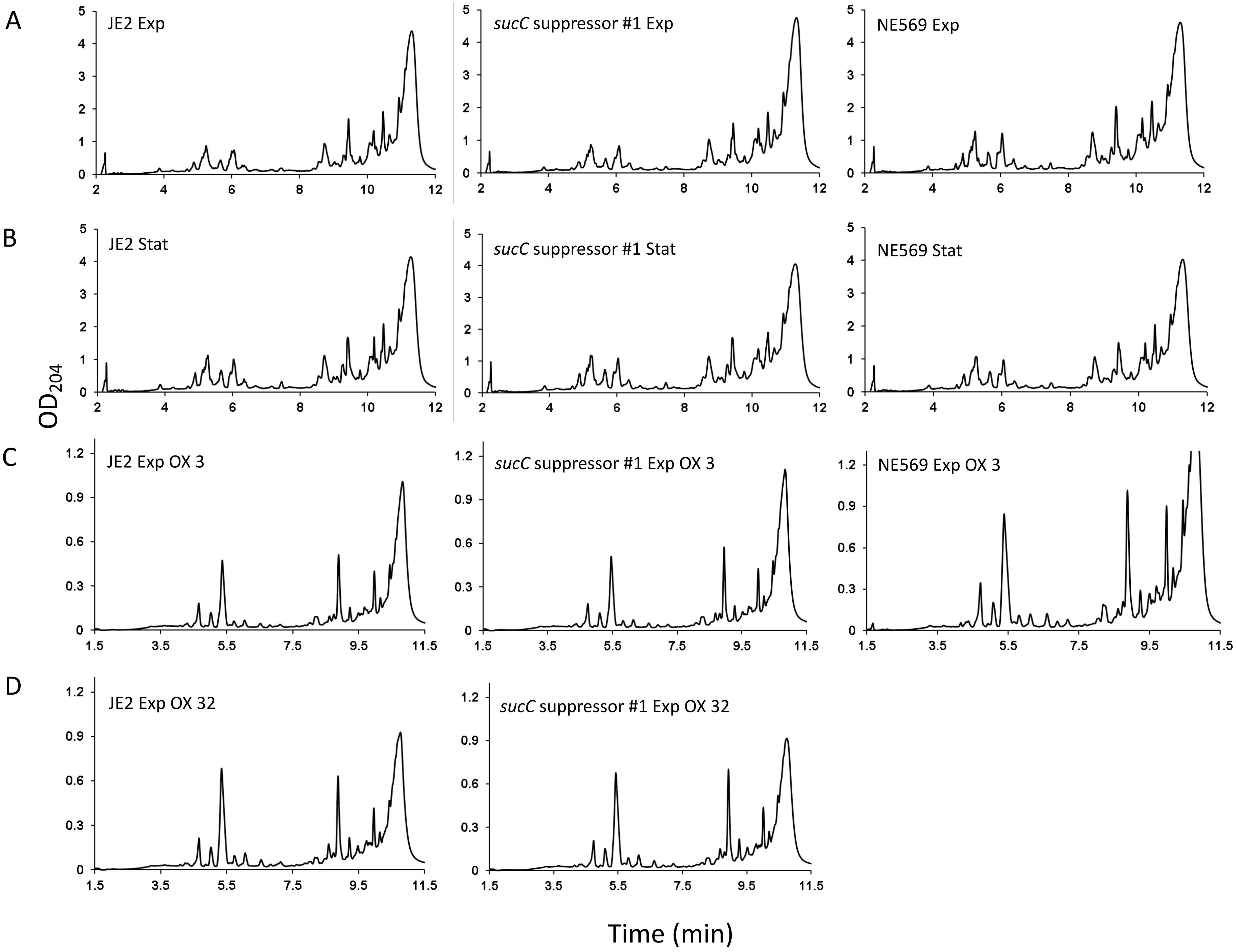
A
B
C
D
JE2 Exp
sucC suppressor #1 Exp
NE569 Exp
JE2 Stat
sucC suppressor #1 Stat
NE569 Stat
JE2 Exp OX 3
sucC suppressor #1 Exp OX 3
NE569 Exp OX 3
JE2 Exp OX 32
sucC suppressor #1 Exp OX 32
OD204
Time (min)

Figure S3. Mutation of *sucC* does not affect PG structure and crosslinking. Representative UV chromatograms of PG extracted from JE2, *sucC* suppressor strain #1 and NE569 (*sucC::*Tn) grown in MHB with oxacillin as indicated. **A.** Exponential phase. **B.** Stationary (Stat) phase. **C.** Exponential phase (Exp) with oxacillin (OX) 3 μg/ml. **D.** Stationary phase with oxacillin (Ox) 32 μg/ml. PG analysis from NE569 (*sucC*) is shown only at 3 mg/ml oxacillin because 32 μg/ml exceeds its MIC. Three biological replicates were analysis and representative chromatograms are shown.
