# Supplementary material for: Accumulation of Succinyl Coenzyme A Perturbs the Methicillin-Resistant Staphylococcus aureus (MRSA) Succinylome and Is Associated with Increased Susceptibility to Beta-Lactam Antibiotics
Source: mBio. 2021 Jun 29;12(3):e00530-21. doi: 10.1128/mBio.00530-21 (PMC8437408; doi:10.1128/mBio.00530-21)
Supplement: FIG S4 [file mbio.00530-21-sf004.pdf]

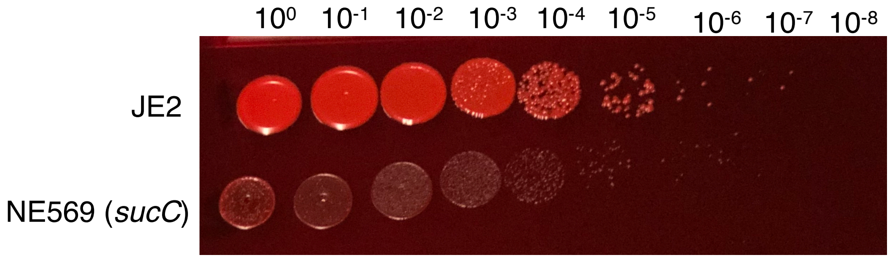

Figure S4. Mutation of *sucC* does not affect susceptibility to Congo Red. Serial dilution of JE2 and NE569 (*sucC*) on MHA supplemented with 0.125% Congo red incubated for 24 h at 37°C. Three biological replicates were analysed and a representative plate is shown.
