# Supplementary material for: Accumulation of Succinyl Coenzyme A Perturbs the Methicillin-Resistant Staphylococcus aureus (MRSA) Succinylome and Is Associated with Increased Susceptibility to Beta-Lactam Antibiotics
Source: mBio. 2021 Jun 29;12(3):e00530-21. doi: 10.1128/mBio.00530-21 (PMC8437408; doi:10.1128/mBio.00530-21)
Supplement: TABLE S1 [file mbio.00530-21-st001.docx]

**Table S1.** Bacterial strains and plasmids used in this study.

| **Strain/Plasmid** | **Relevant details** | | **Source/reference** | | | |
| --- | --- | --- | --- | --- | --- | --- |
| ***Escherichia coli* strain** | | |  | |  |  |
| HST08 | | TaKaRa *E. coli* HST08 Premium Electro-Cells | | TaKaRa | |  |
| ***Staphylococcus aureus* strains** | | |  | |  |  |
| USA300 FPR3757 | | Community associated MRSA isolate of USA300 lineage. SCCmec type IV. Clonal complex 8. | | (4) | |  |
| JE2 | | Plasmid-cured derivative of USA300 LAC | | (28) | |  |
| NE569 | | JE2 *sucC*::Tn. Erm^r^ | | (28) | |  |
| *sucC*::Tn-Kan^r^ | | JE2 *sucC*::Tn. Kan^r^ | | This study | |  |
| *sucC* HoR1 | | HoR mutant of NE569. RelQ Ala_178_Val mutation. | | This study | |  |
| *sucC* HoR2 | | HoR mutant of NE569. RelA Tyr_418_STOP mutation. | | This study | |  |
| *sucC* suppressor #1 | | NE569 suppressor. *sucA* SER_9_STOP mutation. | | This study | |  |
| *sucC* suppressor #2 | | NE569 suppressor. *sucB* Ile_361_Thr mutation. | | This study | |  |
| *sucC* suppressor #3 | | NE569 suppressor. *sucB* 46bp deletion. | | This study | |  |
| NE1770 | | JE2 *sucD*::Tn. Erm^r^ | | (28) | |  |
| NE1724 | | JE2 *pdhA*::Tn. Erm^r^ | | (28) | |  |
| NE1758 | | JE2 *pdhB*::Tn. Erm^r^ | | (28) | |  |
| NE594 | | JE2 *gltA*::Tn. Erm^r^ | | (28) | |  |
| NE861 | | JE2 *acn*::Tn. Erm^r^ | | (28) | |  |
| NE491 | | JE2 *icd*::Tn. Erm^r^ | | (28) | |  |
| NE547 | | JE2 *sucA*::Tn. Erm^r^ | | (28) | |  |
| NE1391 | | JE2 *sucB*::Tn. Erm^r^ | | (28) | |  |
| NE626 | | JE2 *sdhA*::Tn. Erm^r^ | | (28) | |  |
| NE808 | | JE2 *sdhB*::Tn. Erm^r^ | | (28) | |  |
| NE427 | | JE2 *fumC*::Tn. Erm^r^ | | (28) | |  |
| NE1003 | | JE2 *mqo* E1::Tn. Erm^r^ | | (28) | |  |
| NE1381 | | JE2 *mqo* E2::Tn. Erm^r^ | | (28) | |  |
| NE460 | | JE2 *atl*::Tn. Erm^r^ | | (28) | |  |
| NE1714 | | JE2 *relA*::Tn. Erm^r^ | | (28) | |  |
| *sucC*/*sucA* | | *sucC*/*sucA* double mutant. Constructed by transduction of *sucA*::Tn allele from NE547 into *sucC*::Tn-Kan^r^. Erm^r^, Kan^r^ | | This study | |  |
| *sucC*/*sdhA* | | *sucC*/*sdhA* double mutant. Constructed by transduction of *sdhA*::Tn allele from NE626 into *sucC*::Tn-Kan^r^. Erm^r^, Kan^r^ | | This study | |  |
| *sucA*/*relA* | | *sucC*/*relA* double mutant. Constructed by transduction of *relA*::Tn allele from NE1714 into *sucC*::Tn-Kan^r^. Erm^r^, Kan^r^ | | This study | |  |
| RN4220 | | Restriction-deficient *S. aureus*. | | (83) | |  |
| DAR173 | | MRSA; SCC*mec* type IV; Clonal complex 5. | | (29, 30) | |  |
| ATCC 29213 | | MSSA strain for MIC susceptibility testing. | | ATCC | |  |
| ATCC 25923 | | MSSA strain for disk diffusion susceptibility testing. | | ATCC | |  |
| **Plasmids** | |  | |  | |  |
| pLI50 | | *E. coli-Staphylococcus* shuttle vector. Amp^r^ (*E. coli*), Cm^r^ (*Staphylococcus*). | | (84) | |  |
| pKAN | | Plasmid to replace *bursa aurealis* Erm^r^ marker with Kan^r^ marker. Kan^r^, Cam^r^ | | (68) | |  |
| p*sucC* | | pLI50 carrying *sucC* from JE2. Cm^r^ | | This study | |  |
| p*sucCD* | | pLI50 carrying *sucCD* from JE2. Cm^r^ | | This study | |  |
| p*sucD* | | pLI50 carrying *sucD* from JE2. p*sucCD* derivative with 627 bp deletion at 5’ end of *sucC*. Cm^r^ | | This study | |  |
