# Supplementary material for: Accumulation of Succinyl Coenzyme A Perturbs the Methicillin-Resistant Staphylococcus aureus (MRSA) Succinylome and Is Associated with Increased Susceptibility to Beta-Lactam Antibiotics
Source: mBio. 2021 Jun 29;12(3):e00530-21. doi: 10.1128/mBio.00530-21 (PMC8437408; doi:10.1128/mBio.00530-21)
Supplement: TABLE S2 [file mbio.00530-21-st002.docx]

**Table S2.** Oligonucleotide primers used in this study.

| **Target gene** | **Primer name** | **Primer sequence (5’-3’)** |
| --- | --- | --- |
| *sucC* | sucC_F | TACTCAAATCGCCATGCAGC |
|  | sucC_R | AATGACTGAAACCGTTGCCC |
| *sucCD* | sucCD_R | CGCACGACAAATAGCCCATT |
| *mecA* | mecA_F | CATATCGTGAGCAATGAACTGA |
|  | mecA_R | CATCGTTACGGATTGCTTCA |
| *relA* | relA_F | TGGCTTTGCACCTGTTAGAA |
|  | relA_R | TTTTGCCGTCCTGACTTTCA |
| *sucA* | sucA_F | GGCGGTAATGGACTCGGATT |
|  | sucA_R | TCTACGCTATCCCCTACGTT |
| *sdhA* | sdhA_F | TGGGGTGGACTTCAATCTCC |
|  | sdhA_R | TGTGTTTCATGTTGTGGAGTGT |
| **Infusion primers** | |  |
| *sucC* | INF_sucC_F | TCGTCTTCAAGAATTGACGCTTGATAATGCACTG |
|  | INF_sucC_R | TACCGAGCTCGAATTCCTTTACCAGGCGTCACA |
| *sucCD* | INF_sucCD_F | TCGTCTTCAAGAATTTACTCAAATCGCCATGCAGC |
|  | INF_sucCD_R | TACCGAGCTCGAATTCGCACGACAAATAGCCCATT |
| **RT-PCR primers** |  |  |
| *mecA* | mecA1_Fwd | TGCTCAATATAAAATTAAAACAAACTACGGTAAC |
|  | mecA1_Rev | GAATAATGACGCTATGATCCCAA |
| *gyrB* | gyrB_Fwd | CCAGGTAAATTAGCCGATTGC |
|  | gyrB_Rev | AAATCGCCTGCGTTCTAGAG |
