# Supplementary material for: Accumulation of Succinyl Coenzyme A Perturbs the Methicillin-Resistant Staphylococcus aureus (MRSA) Succinylome and Is Associated with Increased Susceptibility to Beta-Lactam Antibiotics
Source: mBio. 2021 Jun 29;12(3):e00530-21. doi: 10.1128/mBio.00530-21 (PMC8437408; doi:10.1128/mBio.00530-21)
Supplement: TABLE S3 [file mbio.00530-21-st003.docx]

**Table S3.** MRM transitions for all metabolites measured in this study.

| **Component Name** | **MRM Transitions** | **Collision energy (V)** |
| --- | --- | --- |
| Oxaloacetate | 131.0 / 87.0 | -9.6 |
| Fumarate | 115.0 / 71.0 | -13 |
| Acetyl-CoA | 808.0 / 79.0 | -150 |
| Succinyl-CoA | 866.0 / 765.4 | -26 |
| Malate | 133.0 / 115.0 | -14.7 |
| Pyruvate | 87.0 / 32.0 | -14 |
| Citrate | 190.9 / 111.1 | -16.1 |
| Isocitrate | 190.9 / 173.0 | -13 |
| Succinate | 117.0 / 99.0 | -14 |
| Phosphoenolpyruvate | 167.0 / 79.0 | -14.6 |
| α-Ketoglutarate | 145.0 / 101.0 | -15 |
| Br-ATP | 585.8 / 79.0 | -120 |
